# A single-cell Raman-based platform to identify developmental stages of human pluripotent stem cell-derived neurons

**DOI:** 10.1073/pnas.2001906117

**Published:** 2020-07-21

**Authors:** Chia-Chen Hsu, Jiabao Xu, Bas Brinkhof, Hui Wang, Zhanfeng Cui, Wei E. Huang, Hua Ye

**Affiliations:** ^a^Institute of Biomedical Engineering, University of Oxford, OX3 7DQ Oxford, United Kingdom;; ^b^Department of Engineering Science, University of Oxford, OX1 3PJ Oxford, United Kingdom

**Keywords:** neural stem cell, Raman spectroscopy, differentiation, machine learning, biomarker

## Abstract

Stem cells with the capability to self-renew and differentiate into multiple cell derivatives provide platforms for drug screening and promising treatment options for a wide variety of neural diseases. Nevertheless, clinical applications of stem cells have been hindered partly owing to a lack of standardized techniques to characterize cell molecular profiles noninvasively and comprehensively. Here, we demonstrate that a label-free and noninvasive single-cell Raman microspectroscopy (SCRM) platform was able to identify neural cell lineages derived from clinically relevant human induced pluripotent stem cells (hiPSCs). By analyzing the intrinsic biochemical profiles of single cells at a large scale (8,774 Raman spectra in total), iPSCs and iPSC-derived neural cells can be distinguished by their intrinsic phenotypic Raman spectra. We identified a Raman biomarker from glycogen to distinguish iPSCs from their neural derivatives, and the result was verified by the conventional glycogen detection assays. Further analysis with a machine learning classification model, utilizing t-distributed stochastic neighbor embedding (t-SNE)-enhanced ensemble stacking, clearly categorized hiPSCs in different developmental stages with 97.5% accuracy. The present study demonstrates the capability of the SCRM-based platform to monitor cell development using high content screening with a noninvasive and label-free approach. This platform as well as our identified biomarker could be extensible to other cell types and can potentially have a high impact on neural stem cell therapy.

## Significance

We developed a label-free and noninvasive single-cell Raman microspectroscopy (SCRM)-based platform to identify neural cell lineages derived from clinically relevant human induced pluripotent stem cells (hiPSCs). Through large-scale Raman spectral analysis, we can distinguish hiPSCs and hiPSC-derived neural cells using their intrinsic biochemical profile. We identified glycogen as a Raman biomarker for neuronal differentiation and validated the results using conventional glycogen detection assays. The parameters obtained from SCRM were processed by a novel machine learning method based on t-distributed stochastic neighbor embedding (t-SNE)-enhanced ensemble stacking, enabling highly accurate and robust cell classification. The platform and the proposed biomarker should also be applicable to other cell types and can shed light on developmental biology and glycogen metabolism disorders.

Neurological disorders are critical causes of mortality, which are reported as the second-leading cause group of deaths, resulting in ∼9.4 million or 16.8% of global deaths (1). In addition to death, these diseases lead to considerable economic costs due to both the treatment cost and productivity loss arising from these diseases. Due to their proven efficacy on cell replacement, neuroprotective effects, activation of endogenous neurogenesis and angiogenesis, and modulation of inflammation and immune responses, neural stem cell (NSC)-based therapies have emerged as a promising strategy for treating neurological diseases which currently lack treatment options (2, 3). NSCs can be derived from human induced pluripotent stem cells (hiPSCs), which have become one of the most appealing cell sources for autologous cell therapy (4, 5). Although considerable research has shown potential clinical applications of NSC therapies for neurological diseases, such as stroke (6), Parkinson’s disease (7), and multiple sclerosis (8), a few major challenges still remain, specifically, identification, isolation, and enrichment of an appropriate and homogeneous population of NSCs prior to transplantation, namely, quality control, which is crucial for clinical practice. A failure to deliver an appropriate cell phenotype could lead to tumorigenesis, failure of host integration, and other pitfalls (9, 10).

To identify cell populations, conventional methods, such as immunochemical staining, fluorescence-activated cell sorting, qRT-PCR, and Western blotting, have been widely used to define and monitor cell differentiation status (11). However, most of these methods require destructive fixation or lysis steps and are generally time consuming (12), limiting their applications toward cell monitoring for clinical transplantation. Other drawbacks include a prerequisite of known selective biomarkers or probes. Depending on the choice of the targets, this might lead to a biased result. In addition, qRT-PCR and Western blotting are unable to capture differences at the single-cell level. Averaging bulk analysis could result in loss of information on cell-to-cell heterogeneity, particularly during the process of cell differentiation (13).

Raman microspectroscopy is a label-free technique which has been applied to study phenotypes of single cells (13–15). It is a nondestructive vibrational spectroscopy based on inelastic scattering of light, reflecting the intrinsic biochemical profiles of cells (16). After Raman detection, individual cells are still intact and viable for subsequent use (17, 18). Previously, single-cell Raman microspectroscopy (SCRM) has been used to discriminate neural progeny in different developmental stages. Raman spectroscopy analysis using principal component analysis (PCA) and linear discriminant analysis were able to discriminate mouse NSCs from glial cells based on a higher concentration of nucleic acids in undifferentiated NSCs (19). Rat hippocampal NSCs have also been examined by Raman spectroscopy to investigate maturation of the developing neural system, where four developmental stages were identified based on PCA (20). Neuronal subtypes, such as excitatory and inhibitory neurons, were also distinguishable through examination of Raman spectroscopy with partial least squares regression-discriminant analysis (12). These previous reports have demonstrated the utility of Raman analysis for phenotypic discrimination in neural cells.

In this study, we developed an in vitro hiPSC-derived neural system and analyzed its biochemical changes using SCRM during the process of differentiation at the single-cell level. In total, we obtained 8,774 single-cell Raman spectra (SCRS) of three different hiPSC lines and their neural derivatives at different developmental stages. By exploiting a generic data analysis pipeline including assessment of intrinsic biomolecules and multivariate visualization via t-distributed stochastic neighbor embedding (t-SNE), we were able to visualize and distinguish different developmental stages of clinically relevant human neural systems. The SCRS analysis revealed a Raman biomarker associated with glycogen to distinguish hiPSCs from NSCs. This result was verified by conventional glycogen detection assays and histology images. We also developed a machine learning classification model based on t-SNE to enhance the efficiency of data analysis and classification for SCRM-based platforms. This SCRM-based classification model has the potential to be extended to other cell types and can be valuable for automated cell phenotype identification and classification for cell sorting, quality control, and quality assurance of clinical-grade cells.

## Results

### Generation and Characterization of Neural Differentiation in hiPSC Cultures.

**Fig. 1.**
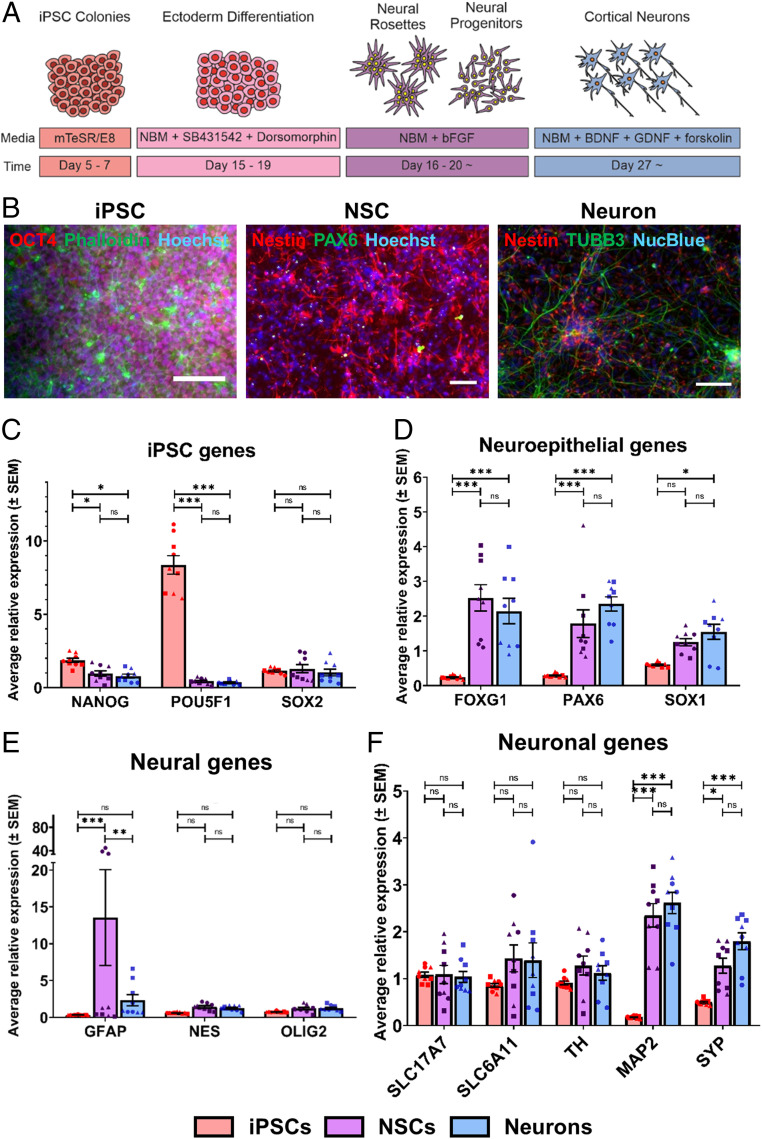
Generation and characterization of hiPSC-derived neural systems. (*A*) Experimental scheme for neuronal differentiation of hiPSCs (NBM, Neural Basal Medium). (*B*) Immunostaining was performed with different protein markers in hiPSCs (*Left*), hiPSC-derived NSCs (*Middle*), and hiPSC-derived neurons (*Right*). (Scale bars, 100 μm.) (*C*−*F*) Gene expression in hiPSCs and hiPSC-derived neural lineages were analyzed by qRT-PCR, including sets of (*C*) pluripotency genes (*NANOG*, *POU5F1*, and *SOX2*), (*D*) neuroepithelial genes (*FOXG1*, *PAX6*, and *SOX1*), (*E*) neural genes (*GFAP*, *NES* and *OLIG2*), and (*F*) neuronal genes (*SLC17A7*, *SLC6A11*, *TH*, *MAP2*, and *SYP*). Circles represent line 010S-1; squares represent line 014S-10; triangles represent line SB-AD3-1. One-way ANOVA with post hoc Tukey’s test was used. All experiments were performed with three biological replicates comprising three technical replicates (*n* = 9). The results represent means ± SEM; * represents *P* < 0.05, ** represents *P* ≤ 0.01, *** represents *P* ≤ 0.001, n.s. = not significant.

The hiPSC cultures (line SB-AD3-1, line 010S-1, and line 014S-10) were differentiated into neuroectoderm by dual-SMAD signaling inhibition when cells reached confluency (Fig. 1*A*) (21). These neural precursors were identified by their self-proliferation and neural rosette structures, characteristic features during hiPSC neural development (22). While expanding hiPSC-derived differentiated neural cultures, the concerns of nonneural cell contamination remained. Previously, polysialic acid-neural cell adhesion molecule (PSA-NCAM) was employed as a marker for purifying neuronal-restricted precursors from neural rosettes (23, 24). By isolating PSA-NCAM−positive NSCs, a highly homogeneous, expandable population of NSCs could be achieved without contamination of undifferentiated iPSCs as well as other undefined derivatives. To monitor neural differentiation in our system, we chose three developmental stages, 1) undifferentiated iPSCs, 2) induced PSA-NCAM−positive NSCs isolated via magnetic-activated cell sorting (MACS), and 3) neurons differentiated from the purified PSA-NCAM−positive NSCs. Cell lineage progression was characterized at different developmental stages using the traditional qRT-PCR method.

Accurate normalization of gene expression data is required to identify differentially expressed genes. Hence, six candidate genes, *ACTB*, *B2M*, *GAPDH*, *HPRT1*, *TBP*, and *YWHAZ*, have been selected, as they have been used as normalizers in previous studies involving stem cells and neuronal differentiation (25–28). Using the geNorm option in qBase, the reference target stability (M value) for these genes was calculated after omitting the least stable gene (*SI Appendix*, Fig. S1*A*). The lowest M value indicated the most stable candidate reference gene. Subsequently, the pairwise variation (V value) was calculated to determine the optimal number of reference genes for normalization in this experiment (*SI Appendix*, Fig. S1*B*). A V value below 0.15 indicates no additional genes need to be included for normalization factor calculations. As such, the optimal normalization factor in this experiment was calculated as the geometric mean of the most stably expressed reference genes *TBP* and *YWHAZ*.

Upon neural differentiation, the cells lost expression of the pluripotency markers, including *NANOG* and *POU5F1* (coding for OCT4), with significantly lower expression levels in the NSCs and derived neurons compared to the iPSCs (Fig. 1*C*). Although *SOX2* is known to be expressed in pluripotent stem cells, it is also expressed in the nervous system at early developmental stages (29). We confirmed that cells were differentiated into neural lineage cells, by identifying the significant increases in neuroepithelial stem cell-related gene expression levels, including *FOXG1*, *PAX6*, and *SOX1* compared to the iPSCs (Fig. 1*D*). The high expression levels of these genes in the differentiated neuron samples indicated that there were undifferentiated NSCs present in the cell culture. We next examined their neural gene expression levels, probing an astrocytic marker, glial fibrillary acidic protein (*GFAP*), a neural marker, *NES*, and an oligodendrocyte marker, *OLIG2* (Fig. 1*E*). While there were trends of higher expression levels of these genes in the NSCs and neurons compared to the iPSCs, only the *GFAP* expression level in the NSCs was significantly higher than that in the iPSCs. It has been reported that a group of radial-glia-like NSCs express *GFAP* and *NES*, generating daughter cells characterized by various proliferation capacities, specific morphology, and their increased neuronal differentiation capacity (30). As astrocytes appear at later developmental stages when neurogenesis decreases in favor of gliogenesis (31), it is likely that the observed higher expression level of *GFAP* originated from the NSCs instead of differentiated astrocytes. Finally, we examined a number of diverse neuronal markers for specific neuron subtypes and mature neurons (Fig. 1*F*). While there were no significant expression differences in neuronal subtype-specific markers (*SLC17A7*, *SLC6A11*, and *TH*), the NSCs and neurons exhibited significantly higher expression levels of mature neuron markers, including *MAP2* and *SYP*, compared to the iPSCs. These data confirmed neuronal differentiation in our system. While there were significant differences in the iPSCs and their neural progenies in general, the NSCs and neurons were more likely to be a mixed coculture of various percentages of NSCs and neurons at different developmental stages.

Since the messenger RNA transcript and protein levels within a sample might not correlate well, we further characterized and validated cell identity at different stages using immunostaining. The hiPSCs expressed an abundance of pluripotency markers, such as NANOG, OCT4, and SOX2 (Fig. 1*B* and *SI Appendix*, Fig. S2*A*) (21). The transcription factor PAX6 and Nestin, an intermediate filament protein, are key regulators of NSC self-renewal and neurogenesis (30). For characterization, cells expressing PAX6 and/or Nestin were regarded as hiPSC-derived NSCs. These cells were maintained at the proliferative stage to keep their differentiation potential and could be further differentiated in response to specific stimuli. After neuronal differentiation for 2 wk, the populations of hiPSC-derived neurons were examined with immunostaining of βIII-tubulin (TUBB3), which is a structural protein expressed in neurons contributing to microtubule stability in cell bodies and axons (32). While NSCs could be identified by Nestin expression, differentiated neurons were recognized by expression of TUBB3 (Fig. 1*B*).

In addition to the gene and protein expression of differentiation and maturation markers, whether a neuron is able to develop synaptic activities and further generate action potentials is critical during neural development (33). Therefore, we employed Ca^2+^ imaging to examine the functionality of the generated neurons (*SI Appendix*, Fig. S2 *B* and *C*). After 7 wk of differentiation, the differentiated neurons could generate and transmit calcium transients by spontaneous activity, indicating that the derived neurons in our system were functionally active and able to simulate their in vivo compartments. Similar to the qRT-PCR results, the immunostained NSC and neuron populations exhibited a developmental transition with the presence of both self-renewing, multipotent NSCs and differentiated neurons. Assessment of Ca^2+^ imaging demonstrated the hiPSC-derived neurons acquired physiological function over cell maturation, and our cell model system was capable of recapitulating representative characteristics at different stages of neural development.

### Raman Profiling of hiPSC-Derived Neural Cells.

**Fig. 2.**
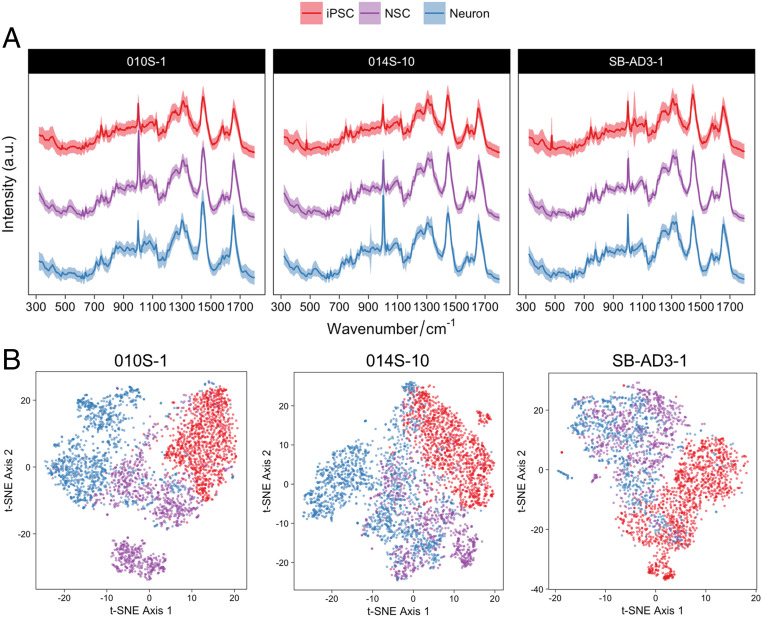
Identification of Raman signatures in the hiPSC-derived neural system from three different hiPSC lines. (*A*) Averaged SCRS (*n* = 8,774) acquired from the hiPSCs (*n* = 3,316), NSCs (*n* = 2,342), and neurons (*n* = 3,116) from different hiPSC lines. (*B*) Multivariate visualization of the SCRS via t-SNE depicts the differences between hiPSCs and their neural lineages. (The red, purple, and blue colors represent iPSCs, NSCs, and neurons, respectively.)

After the generation and characterization of cells at different developmental stages, we examined the potential of using the label-free SCRM technique to study single-cell phenotypes using their intrinsic biochemical profiles. Three undifferentiated hiPSC lines and their neural derivatives were examined, and their SCRS were pooled and analyzed (Table 1 and Fig. 2*A*).

**Table 1.** Numbers of SCRS obtained for the three developmental stages during neuronal differentiation in hiPSCs

Cell lines	iPSC	NSC	Neuron	Total no. of SCRS
010S-1	1,026	979	1,128	3,133
014S-10	1,117	697	1,513	3,327
SB-AD3-1	1,173	666	753	2,592
Total no. of SCRS	3,316	2,342	3,116	8,774

Note: All experiments were performed with three biological replicates comprised of three technical replicates; *n* = 8774 spectra in total.

The fingerprint region (320 cm^−1^ to 1,800 cm^−1^) of an SCRS captures most of the vibrational modes of biomolecules within a single cell, representing the unique observable characteristics or Raman phenotype of a cell. The fingerprint region contains 443 Raman bands (based on a spectral resolution of ∼3.3 cm^−1^ in a spectral range of 320 cm^−1^ to 1,800 cm^−1^) in current acquisition settings. Due to the high dimensionality of SCRS, multivariate dimension-reducing techniques, such as PCA and t-SNE algorithms, were used to highlight the intrinsic differences and to visualize the data in a lower dimension. We have compared the visualization results of SCRS by t-SNE and PCA and found a much better differentiation of t-SNE for depicting differences among hiPSC-derived neural lineages (*SI Appendix*, Fig. S3). The t-SNE plots combing three different cell lines with three technical replicates in each cell line (8,774 spectra in total) are shown in Fig. 2*B*. The t-SNE plots permitted us to distinguish, to some extent, between the datasets of the hiPSCs and the hiPSC-derived neural progenies. This shows the ability of SCRS and t-SNE to discriminate cell populations at different developmental stages, particularly hiPSCs and their neural derivatives.

While clusters of hiPSCs are relatively tight and homogeneous (especially in cell lines 010S-1 and 014S-10), high cellular heterogeneity was observed in the hiPSC-derived neural progenies at the single-cell level. A certain degree of overlap between the clusters of hiPSC-derived NSCs and neurons in the t-SNE score plots correlated well with the results previously acquired by qRT-PCR and immunostaining, confirming that the cell populations in these samples are likely to be a mixed coculture of NSCs and neurons, where cells matured at different speeds. This suggests a great level of single-cell heterogeneity during stem cell differentiation, highlighting the importance of single-cell−level analysis for characterization and quality control.

### Identification of Potential Biomarkers for Cell Quality Control.

**Fig. 3.**
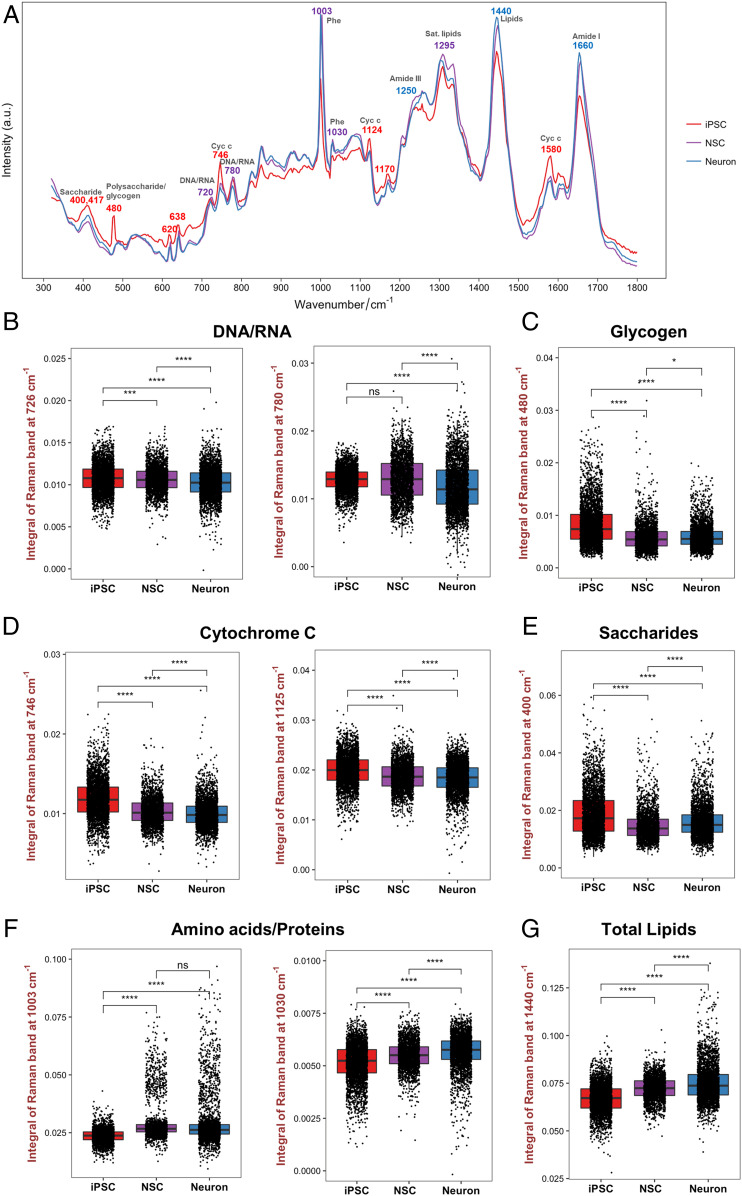
Identification of potential biomarkers using SCRS. (*A*) Average Raman spectra acquired from the hiPSCs (red), NSCs (purple), and neurons (blue) from three different hiPSC lines annotated with Raman bands identified in cell lineage commitment. (*B*−*G*) Comparisons of the Raman band intensity between different cell populations were quantified for (*B*) nucleic acids (726 and 780 cm^−1^), (*C*) glycogen (480 cm^−1^), (*D*) cytochrome C (746 and 1,125 cm^−1^), (*E*) saccharides (400 cm^−1^), (*F*) proteins (1,003 and 1,030 cm^−1^), and (*G*) total lipids (1,440 cm^−1^). One-way ANOVA with post hoc Tukey’s test was used. All experiments were performed with three biological replicates comprising three technical replicates with 8,774 Raman spectra acquired from the hiPSCs (*n* = 3,316), NSCs (*n* = 2,342), and neurons (*n* = 3,116). The results represent means ± SEM; * represents *P* < 0.05, *** represents *P* ≤ 0.001, **** represents *P* ≤ 0.0001.

During the progression of neural differentiation, several changes in Raman bands for major cellular components and metabolites were observed (Fig. 3*A*). Similar to the t-SNE visualization, the most prominent differences were found between the SCRS of hiPSCs and their neural lineages, including Raman bands at 400 and 417 cm^−1^ (saccharides), 480 cm^−1^ (glycogen), 746 cm^−1^, 1,125 cm^−1^, and 1,580 cm^−1^ (cytochrome *c*), 720 and 780 cm^−1^ (DNA/RNA), 1,003 and 1,030 cm^−1^ (phenylalanine), 1,295 and 1,440 cm^−1^ (lipids), and 1,660 cm^−1^ (proteins) (Table 2).

**Table 2.** Assignment of specific Raman bands to vibrational modes and biological molecules

Raman Wavenumber (cm^−1^)	Biomolecule assignment	Molecular vibration	Refs.
400	Saccharides	Skeletal modes of carbohydrates	(34)
417	Saccharides	β(COC); β(CCC), β(CCO), β(OCO)	(34)
480	Glycogen	ν(C1−O−C4); Ring breathing	(34)
720	Adenine	Ring breathing	(35)
746	Cytochrome C	ν(pyr breathing); ν(C−C)	(36)
780	DNA/RNA	Ring breathing of C, T and U	(35)
1,003	Phenylalanine	Phe symmetric ring breathing	(35)
1,030	Phenylalanine	C−H in plane bend	(35)
1,124	Cytochrome C	ν(C−N)	(36)
1,250	Proteins	Amide III	(37)
1,440	Lipids	δ(CH_2_, CH_3_)	(38)
1,660	Proteins	Amide I	(37)

In order to confirm the variations of biomolecules at different cell stages, we further performed semiquantification of biomolecules by integrating relevant Raman band areas (Fig. 3 *B*–*G*). Some of the SCRS features revealed that structural differences between neuron chromatin and iPSC/NSC chromatin were evident at this level of analysis. The intensity of 780 cm^−1^ nucleic acid band, resulted from the ring-breathing modes of uracil (U), cytosine (C), thymine (T), and the O−P−O stretching, was significantly lower in neurons compared to iPSCs (*P* ≤ 0.0001) and NSCs (*P* ≤ 0.0001) (Fig. 3*B*). While there was no significant difference between iPSCs and NSCs in 780 cm^−1^ intensity, NSCs exhibited a significantly lower intensity at 726 cm^−1^ compared to iPSCs. These bands likely reflect changes in the content of nucleic acids and can relate to changes in the cell cycle. The bands at 780 cm^−1^ and 1,440 cm^−1^ showed an increased standard deviation in the differentiated neuronal progenies. As the production of DNA/RNA and lipids is associated with cell cycle regulations, this could imply an increased heterogeneity of cell phenotypes across neural development, where the differentiated neuronal cells do not proliferate and the cell cycle is arrested.

The Raman band intensity of polysaccharides/glycogen at 480 cm^−1^ was significantly decreased following the progression of neural differentiation (Fig. 3*C*). The results also showed that iPSCs exhibited significantly more cytochrome *c* (bands at 746 and 1,125 cm^−1^) compared to NSCs and neurons (Fig. 3*D*), and there was a significantly higher intensity of the saccharide band at 400 cm^−1^ in the iPSCs (Fig. 3*E*). Protein-related band intensities at 1,003 cm^−1^ (i.e., phenylalanine symmetric ring breathing) and 1,030 cm^−1^ (CH2/CH3 bending modes) were significantly lower in the undifferentiated iPSCs (Fig. 3*F*). A noticeable difference was observed at 1,440 cm^−1^ lipid (i.e., C−H deformation), where there was a significant increase in differentiated neural lineage cells in the progression of differentiation (Fig. 3*G*).

**Fig. 4.**
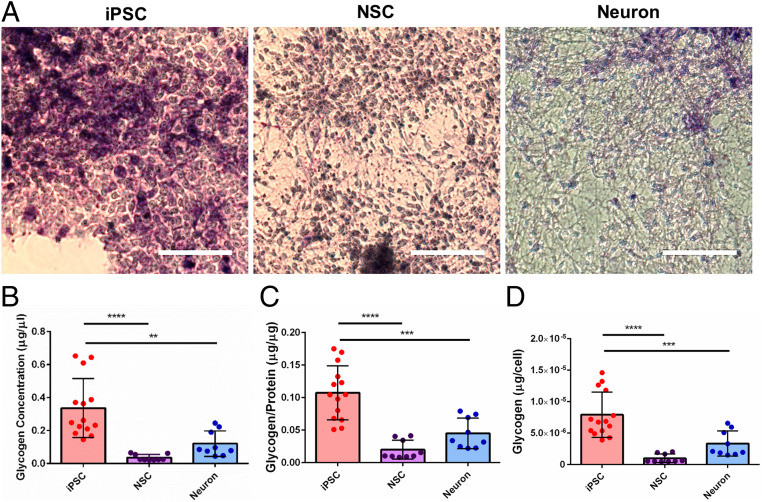
Confirmation of glycogen as a potential biomarker for cell quality control and lineage specification. (*A*) PAS staining of hiPSCs, hiPSC-derived NSCs, and hiPSC-derived neurons is shown in magenta color and counterstained with hematoxylin in deep blue-purple color. (Scale bars, 100 μm.) (*B*−*D*) Quantitative comparisons of the glycogen concentration in different cell lineages using a commercially available glycogen detection assay. One-way ANOVA with post hoc Tukey’s test was used. All experiments were performed with three biological replicates comprising at least three technical replicates (iPSC: *n* = 14; NSC: *n* = 9; neuron: *n* = 9). The results represent means ± SEM; ** represents *P* ≤ 0.01, *** represents *P* ≤ 0.001, **** represents *P* ≤ 0.0001, and there was no statistical significance between the other groups.

Since there is still a lack of information on the energy metabolism during neuronal differentiation in stem cells, we were particularly interested in the significant decrease of glycogen Raman band intensity found in differentiated neural progenies compared to iPSCs. Due to immature and inactive mitochondria at the stage of iPSCs, it is likely that glycolysis is the main source of energy for iPSC proliferation and the initiation of differentiation (39, 40). Upon neural differentiation, glycogen runs out and mitochondria become functional, regulating the metabolic transition to oxidation and the shift of the source of energy (39). To validate our SCRS results, we performed periodic acid−Schiff (PAS) histological staining, which is commonly used to detect glycogen deposits. Evidently, the immunohistochemical staining exhibited a higher intensity of the magenta color in iPSCs compared to NSCs and neurons, indicating that more glycogen was present in the iPSCs (Fig. 4*A*). We also confirmed our results with a commercially available glycogen detection assay (Fig. 4 *B*–*D*). Our results showed that iPSCs acquired significantly higher glycogen concentrations (iPSC: 0.336 ± 0.048 μg/μL; NSC: 0.037 ± 0.006 μg/μL; Neuron: 0.121 ± 0.026 μg/μL) as well as significantly higher glycogen content normalized to both protein content (iPSC: 0.107 ± 0.011 μg/μg protein; NSC: 0.020 ± 0.005 μg/μg protein; Neuron: 0.045 ± 0.008 μg/μg protein) and total cell number (iPSC: 7.925 ± 0.960 pg per cell; NSC: 0.984 ± 0.228 pg per cell; Neuron: 3.339 ± 0.668 pg per cell). These population-based results are consistent with the single-cell Raman analysis. Collectively, the study demonstrates the potential of glycogen as a reliable biomarker to discriminate cells in different neural developmental stages for quality control and lineage differentiation.

### Comparative Study of Cells Derived from Three hiPSC Lines.

The hiPSC technology provides an invaluable platform for the development of patient-specific cell sources for disease modeling and regenerative therapies. In addition to the intrinsic variability between different subjects, genetic and epigenetic variations in iPSCs have also been reported during iPSC generation and maintenance (41). We looked into the differences between different cell lines using the previous qRT-PCR and immunostaining image analysis (*SI Appendix*, Fig. S4, and the results for statistical analyses of qRT-PCR are shown in *SI Appendix*, Figs. S5–S8). For the gene expression levels, cells at the same developmental stages possessed similar profiles among three different cell lines (*SI Appendix*, Figs. S4 *A*–*D* and S5–S8). Although NSCs from line 010S-1 exhibited a lower gene expression level for *FOXG1* and significantly higher expression levels for *GFAP* (*SI Appendix*, Figs. S4 *B* and *C*, S6, and S7), there was no noticeable difference between the expression levels of neuronal genes among different cell lines (*SI Appendix*, Figs. S4*D* and S8). To verify the gene expression data, we also examined the cell line differences in protein expression level via image analysis of immunostaining, particularly focusing on specific cell markers related to neuronal differentiation and NSC proliferation. We analyzed the differences in the percentage of βIII-tubulin^+^ cells and the percentage of Nestin^+^ cells in the total cell population after neuronal differentiation for 2 wk (*SI Appendix*, Fig. S4 *E* and *F*). There was no significant difference in neuronal differentiation between the three cell lines. Interestingly, line SB-AD3-1 exhibited a significantly higher proliferative Nestin^+^ cell population (55.2 ± 4.3%) compared to line 010S-1 (24.0 ± 3.2%) and line 014S-10 (22.5 ± 2.8%).

**Fig. 5.**
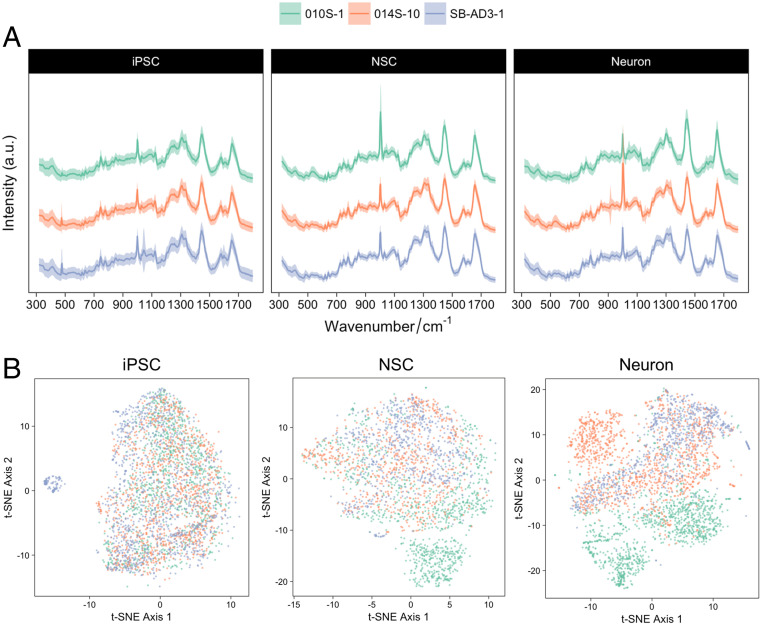
Comparison of Raman signatures in neural systems derived from different hiPSC lines. (*A*) Comparison of Raman spectra of cell lineages from various hiPSC lines, including line 010S-1 (green; *n* = 3,133), line 014S-10 (orange; *n* = 3,327), and line SB-AD3-1 (lavender; *n* = 2,592). (*B*) Multivariate analysis of the Raman spectra of different hiPSC lines at various differentiation stages. (The green, orange, and lavender colors represent lines 010S-1, 014S-10, and SB-AD3-1, respectively.)

We further explored the differences between cell lines derived from different subjects at the single-cell level, by SCRS visualization using t-SNE (Fig. 5). Overlapping clusters were observed between hiPSCs derived from three different cell lines. However, with the progression of neural differentiation, while there were still overlapping spectra, the variances between cell lines increased. While iPSCs were maintained in a synchronized pluripotent status, after neural induction, the cells were primed for differentiation with heterogenous time schemes. Although we have preselected the NSC populations with MACS using PSA-NCAM, the difference in the degrees of variance, particularly in the differentiated neuronal populations, could result from the intrinsic variance in the rate of differentiation and maturation between different cell lines. It is worth noting that the results from SCRS analysis could provide a more comprehensive and detailed analysis, revealing differences in the cells’ biochemical profiles compared to targeted, biased gene or protein biomarkers.

### Machine Learning Classification Models.

In the previous *Results* sections, we indicated that iPSCs and their derived neural progenies could be distinguished based on their distinct phenotypic SCRS. Besides feature extraction from SCRS to find informative biovariables, classification based on their spectra is often desirable for diagnostic purposes. As manual generic data analysis could be difficult and time consuming when handling a complex problem or a large and complex dataset, we explored the application of machine learning in constructing classification models to classify different developmental stages of cells based on their SCRS.

A total of 8,774 spectra were divided into a training set (*n* = 6,581 spectra) and a testing set (*n* = 2,193) to evaluate the performance of a particular model. A number of classifiers were constructed and evaluated (*SI Appendix*, Table S2). Previous studies using t-SNE−transformed datasets in building a classification model have found significant improvement in its model performance, especially in the field of medical sciences (42–44). Here, we also introduced t-SNE to preprocess our SCRS datasets and used the outputs of t-SNE as additional inputs into different classifiers. Performances of various models with the original datasets and the t-SNE−transformed datasets were listed and compared (*SI Appendix*, Table S2). The best model performance was achieved in a t-SNE−transformed stacked stochastic gradient boosting (SGB) model, in which a high accuracy of 97.5% was reached. In this model, the SCRS dataset was initially transformed by t-SNE into a lower-dimensional space. Then, five models were trained individually, namely k-Nearest Neighbor (kNN), SGB, random forest (RF), linear support vector machine (SVM), and SVM with radial basis function (RBF) kernel. Five learning curves that correspond to individual models were plotted to illustrate the effectiveness of the size of the dataset (8,774 spectra/536 cells) (*SI Appendix*, Fig. S9*A*). A different data splitting method, which splits the dataset by three biological replicates, was also evaluated to achieve better clinical relevance. The comparison between the two data splitting methods is summarized in *SI Appendix*, Fig. S9*B*. These models were subsequently combined to train a stacking classification model to achieve better classification power and accuracy by using the output of these models as inputs into another SGB algorithm.

Table 3 presents the classification results of the stacked model in classifying the independent testing set (*n* = 2,193) which did not participate in the process of training the model. The performance of the classification test achieved a sensitivity of 98.7%, 95.8%, and 97.2% for iPSCs, NSCs, and neurons, respectively, and a specificity of 99.5%, 98.6%, and 98.2% for iPSCs, NSCs, and neurons, respectively (Table 3). The overall accuracy rate is as high as 97.5%. Generally, high sensitivity usually comes at the expense of reduced specificity with more false positives, and, vice versa, high specificity accompanies lower sensitivity with more false negatives. In our case, specificity is more important for the quality control of stem cell transplants, where no single undifferentiated pluripotent stem cell should be transplanted into the human body to reduce or eliminate the risk of developing tumors. With the use of the SCRS platform in combination with the stacked classification model, we were able to achieve high sensitivity, high specificity, and high accuracy of cell classification during different stages of neural development.

**Table 3.** Classification of hiPSCs and hiPSC-derived neural lineage cells by the ensemble machine learning model (overall accuracy at 97.5%)

	Ground truth		
	iPSC	NSC	Neuron
Model prediction			
iPSC	587	4	3
NSC	4	530	19
Neuron	7	19	750
Sensitivity (%)	98.7	95.8	97.2
Specificity (%)	99.5	98.6	98.2

## Discussion

This study employs Raman spectral analysis to distinguish cells from different cell donors and various differentiation stages in clinically relevant human neural systems. The SCRS of undifferentiated iPSCs and their neural lineage descendants showed distinct characteristics, and different cell phenotypes were visualized as clear clusters using t-SNE multivariate analysis. These results, along with noninvasive Raman-activated cell sorting techniques (45), such as the recent development of a high-throughput Raman flow cytometer (17, 46), demonstrate the potential of transforming our platform into a noninvasive and label-free cell sorting platform to facilitate clinical uses of stem cell-related interventions and therapies. While biomolecular profiles of Raman spectra are complex, it can be challenging and time consuming to collect, process, and analyze a huge number of data manually. To transform this method into a faster and more efficient process, we explored the application of machine learning to process and interpret our Raman data, where a machine learning classification model was built upon the t-SNE−transformed SCRS using ensemble learning of various classifiers.

Our SCRS analysis indicated that differentiated hiPSCs (i.e., NSCs and neurons) were clearly dominated by protein (1,003, 1,030, 1,250, and 1,660 cm^−1^) and lipid bands (1,295 and 1,440 cm^−1^), similar to a previous report indicating that spontaneously differentiated human pluripotent stem cells acquired dominating protein and lipid bands, and these changes could be attributed to changes in cellular metabolism (47). It is known that aromatic amino acids (e.g., tryptophan, tyrosine, and phenylalanine) are precursors for neurotransmitters, such as dopamine, serotonin, and norepinephrine, which play major roles in transducing chemical signals among neurons and their target cells (48). The increased protein contents, particularly phenylalanine and other aromatic amino acids, could be regulated by intrinsic biosynthesis pathways for supporting cell lineage transitions from pluripotent cells into appropriate neuronal identities. We identified a decrease in the nucleic acid bands (726 and 780 cm^−1^) in the differentiated neural progenies. Previously, a similar decrease in the cytoplasmic RNA concentration was observed using Raman spectroscopy when mouse neural progenitor cells differentiated into glial cells (19). It is known that neuroepithelial progenitor cells in the ependymal layer initially build up a high level of cellular RNA content at early stages of differentiation (49). However, as the cells progress toward the endpoint of differentiation, the RNA content drops distinctly. The accumulation of RNA is usually associated with protein synthesis activity. Presumably, cells in the differentiated states are no longer involved in a significant level of protein synthesis. Small wavenumber shifts were present between iPSCs and their neural progenies, including the 1,003 cm^−1^ phenylalanine band. It has been suggested that differences in recurrent protein secondary structures might result in a shift of Raman bands (50). For example, the phenylalanine bands shifted between 997 cm^−1^ and 1,007 cm^−1^ in different types of collagen, and similar structural motifs also occur in actin (51). During neural development, cytoskeletons, including actin filaments and microtubules, dynamically remodel to adopt morphological transitions or to drive cell migration and the development of axons, dendrites, and their branches (52). It is possible that the observed shifts demonstrate the structural remodeling of cytoskeletal proteins, which possess drastic changes during neural development. There was significantly more cytochrome *c* present in hiPSCs (bands at 746 and 1,125 cm^−1^) compared to NSCs and neurons. It was known that single-cell dissociation of human pluripotent stem cells disrupts E-cadherin−mediated cell−cell interactions, causing ROCK-dependent hyperactivation of actomyosin network, resulting in cell apoptosis where cytochrome *c* was released from mitochondria (53). The observed up-regulated cytochrome *c* in hiPSCs could result from the effects of single-cell dissociation. Future work incorporating ROCK inhibitors before cell characterization using SCRS might reveal detailed regulations of cytochrome *c* during neural development.

Glycogen, a storage form of glucose, acting as the principal energy source rapidly available for several organs, exhibits at low concentrations in the brain (54). Previously, it was reported that hiPSCs acquire a prevalent glycolytic state compared to differentiated fibroblasts (39). Although characterization of neural development in hiPSCs has been widely explored using genome-wide profiling and biochemical analysis (55–57), there is very little research conducted focusing on the changes related to cellular metabolism, and none of them were performed at the single-cell level with a quantitative measure (58). Our study demonstrates that SCRM is able to provide semiquantitative glycogen content in situ at the single-cell level (59). The SCRS results revealed that hiPSCs possessed significantly more glycogen (480 cm^−1^), which was consistent with the results acquired from PAS histological staining and commercially available glycogen detection assays. As the change in glycogen content was significant in both SCRS and conventional assays, we suggest that glycogen can be used as a biomarker to distinguish hiPSCs from their differentiated neural progenies. Previously, evidence of glycogen variations has been reported during maintenance of human embryonic stem cells (hESCs) and hESC differentiation using Raman microspectroscopy (60–63). While glycogen increased during hESC differentiation into pancreatic insulin-positive cells (60), interestingly, our results demonstrated a different trend where a decrease of glycogen was found in differentiated neural lineages. These inconsistent results on the glycogen concentration were more likely attributed to different cell lineages instead of differences between hESCs and hiPSCs (47, 64), suggesting the importance of tracking the progression of lineage-specific differentiation and acknowledging the differences among cell types.

Glycogen serves as one of the major forms of energy source in vivo; however, with respect to cellular glycogen metabolism, there is very little information on this important topic over the course of stem cell differentiation. Conventional methods to assess glycogen storage and turnover include electron microscopy, histochemical PAS reaction, immunostaining with specific antibodies against glycogen, or biochemical methods analyzing glycogen contents in cell or tissue homogenates via degradation or hydrolysis of glycogen (40). However, each of these techniques has drawbacks. While electron microscopy highly relies on the expertise and is both time consuming and costly, immunocytochemical techniques generally require sample fixation, which might result in up to 70% glycogen loss due to its predominantly soluble form in the cytoplasm (65). Glycogen biochemical assays require a certain amount of sample and a range of tedious steps with oxidation and glucose background subtraction, making them challenging and confounding if proper background controls are not included in the assay. Most important of all, all these methods require cell fixation or lysis, which makes them not translational for clinical applications. SCRS acquires the advantage of detecting glycogen directly as well as noninvasively, and it has previously been used to quantify absolute glycogen contents in situ (59). Although the cells were fixed before examination in this study, it has been demonstrated that live cells could be used in similar studies (66–68). To fulfill the need of live cell characterization and detection in clinical settings, in the future, the phototoxicity of the Raman laser irradiation on cell viability and functionality should be evaluated. A longer wavelength of laser can also be employed to avoid potential cell damage (69). With this platform, we can probe glycogen content with a more feasible and accurate approach in real time and can potentially provide insights into glycogen metabolism in live cells at the single-cell level.

It is known that the equilibrium between utilization of glucose and glycogen synthesis is regulated by the phosphatidylinositol-3-kinase (PI3K)-AKT signaling pathway via glycogen phosphorylase and glycogen synthase (70). Although there are many aspects that await elucidation, an earlier study indicated that glycogen synthesis in human pluripotent stem cells could be modulated by differentiation-dependent bone morphogenetic protein 4-related pathways or differentiation-independent and pluripotent state-dependent glycogen synthase kinase 3 (GSK-3)-related pathways (71). The equilibrium between glycogen accumulation and glycogenolysis in pluripotent stem cells was proposed as a metabolic switch, which can further regulate pluripotent state transitions in stem cells as well as cell differentiation. Although it is well known that glucose is the main energy source in the brain and glycogen generally is not present in neurons but only present in astroglial cells (54), the changes of glycogen metabolism over neuronal differentiation remain unexplored. GSK-3, a serine/threonine protein kinase with two isoforms, GSK-3α and GSK-3β, and having the ability to phosphorylate glycogen synthase and regulate glycogen metabolism, is able to regulate neural cell differentiation (72). GSK-3 inhibition activates the Wnt/β-catenin pathway, enhancing proliferation and expansion of neural progenitors and suppressing neuronal differentiation. In contrast, Wnt3a has been indicated to increase neurogenesis independent of the Wnt/β-catenin transcriptional activity (73). In the central nervous system, GSK-3β is developmentally regulated, but its role in regulating neural differentiation remains controversial. While GSK-3β was shown to facilitate neurite outgrowth by preventing E2F1 from inhibiting the transcription of cyclin-dependent kinase inhibitors p21 and p15 (74), inhibition of GSK-3β also resulted in the expression of nonphosphorylated collapsin response mediator protein 2 and the enhancement of axonogenesis and axon growth (75). Furthermore, accumulation of glycogen or glycogen-like inclusions has been reported in several neurological diseases, such as Parkinson’s disease, Huntington’s disease, and Lafora disease (76). With the proven feasibility and accuracy of Raman spectroscopy, this approach would be of considerable benefit for investigating and elucidating the correlations between glycogen levels not only during neural development but also during neurodegeneration in the future.

In this study, we are particularly interested in the quality control of stem cell transplants for treating neurological diseases. It has been shown that transplantation of early-stage NSCs or immature neurons resulted in the greatest neural tissue and functional repair compared to transplantation of more mature neurons (77). Thus, we chose to examine undifferentiated hiPSCs, NSCs, and neurons differentiated for 2 wk (immature neurons) in this study. We explored the use of machine learning methods for automated identification of cells’ developmental stages based on their SCRS. By acquiring 8,774 SCRS of iPSCs, NSCs, and neurons, different classification models were constructed and compared. By training a t-SNE−transformed stacked model, a high accuracy rate of 97.5% was achieved. For the purpose of stem cell therapy, it is of the greatest importance to avoid transplantation of any undifferentiated iPSCs into the human body. Although it is nonessential to distinguish NSCs from neurons in clinically relevant settings, it is technically feasible to include distinct mature neurons with functional synapses (generally differentiated for more than 7 wk from NSCs) to understand the properties of mature neurons and their differences from these earlier stages of neural cells (78). It is also worth noting that, in the present study, we used machine learning models for classification, where the original data were preprocessed and inputted manually. However, we have further established a complete pipeline which includes automated data processing as well as cell classification to maximize the automation.

Our SCRM platform demonstrates the potential of classifying neural developmental stages of clinically relevant human pluripotent stem cells with high accuracy and automation and is extendable to other cell types. Probing the biomarker, glycogen, using SCRS could also provide valuable insight into neural development as well as an understanding of glycogen-related neurological diseases.

## Materials and Methods

### Cell Culture.

The hiPSC lines, line SB-AD3-1 (from a 32-y-old female healthy subject), line 010S-1 (from an 18-y-old female healthy subject), and line 014S-10 (from a 15-y-old male healthy subject) were used in the study. The cells were maintained on Matrigel (Corning)-coated culture plates with the use of mTeSR1 media (STEMCELL Technologies) and Essential 8 media (Thermo Fisher Scientific) and were passaged using 0.5 mM (ethylenedinitrilo)tetraacetic acid (EDTA; pH 8.0; Thermo Fisher Scientific) in sterile phosphate buffer solution (PBS) when they reached 80 to 90% confluence. Neural differentiation was based on previously published protocols (33, 79, 80). Briefly, hiPSCs were used for neural conversion when they reached confluency. Neural Basal Medium was prepared by mixing 1:1 ratio of [Advanced DMEM/F-12 medium (Thermo Fisher Scientific), 1% vol/vol N-2 supplement (Invitrogen), 0.2% vol/vol B27 Supplement (Invitrogen), 1% vol/vol GlutaMAX (Invitrogen), 1% vol/vol penicillin/streptomycin (Invitrogen)] and [Neurobasal Medium (Thermo Fisher Scientific), 2% vol/vol B27 Supplement (Invitrogen), 1% vol/vol GlutaMAX (Invitrogen), 1% vol/vol MEM Non-Essential Amino Acids (Thermo Fisher Scientific), 1% vol/vol penicillin/streptomycin (Invitrogen)]. The cells were differentiated via dual SMAD signaling inhibition (21), using neural induction medium [Neural Basal Medium supplemented with SB431542 (10 μM; Calbiochem) and InSolution AMPK Inhibitor, Compound C (2 μM; Calbiochem)] for 7 d to 10 d. After enzymatic dissociation, NSCs were passaged and plated down on laminin from mouse sarcoma basement membrane (Sigma-Aldrich)-coated plates in the Neural Basal Medium. After 3 d to 5 d, hiPSC-derived NSCs proliferated and formed neural rosette structures, and the cell culture medium was changed into F20 Medium [Neural Basal Medium supplemented with 20 ng/mL Recombinant Human FGF-basic (PeproTech)]. NSCs were passaged every 5 d to 7 d on laminin for the first two to three passages and on Matrigel for later passages. Further differentiation of the NSCs into neurons was performed in Neural Basal Medium supplemented with 10 ng/mL brain-derived neurotrophic factor (PeproTech), 10 ng/mL glial cell-derived neurotrophic factor (PeproTech), and 10 μM forskolin (Sigma-Aldrich).

### RNA Extraction, Reverse Transcription, and qPCR.

Samples were collected after 2 d in culture for iPSCs and NSCs or 14 d after differentiation initiation for the neurons. Cell pellets were lysed and stored at −80 °C in TRIzol reagent (Thermo Fischer Scientific) until total RNA was extracted as described before (26). In short, 1-bromo-3-cholopropane (Sigma-Aldrich) was used to extract the RNA. Subsequently, the RNA was purified with phenol/chloroform/isoamyl alcohol (125:24:1, Sigma-Aldrich) and precipitated with 2-propanol (Sigma-Aldrich). After washing in 75% EtOH, the RNA pellet was dissolved in RNase/DNase free water (Thermo Fisher Scientific). RNA quantity was determined using a Nanodrop One spectrophotometer (Thermo Fisher Scientific) before quality assessment on an Agilent 2100 bioanalyzer (Agilent Technologies). All measurements indicated intact and good-quality RNA with RNA integrity numbers greater than 9. The QuantiTect Reverse Transcription kit (Qiagen) was used to convert 0.5 μg to 1 μg of RNA into complementary DNA (cDNA) per manufacturer’s instructions, including a genomic DNA wipe-out step. Final cDNA sample volumes of 20 µL were diluted fourfold in RNase/DNase free water and stored at −20 °C until used for qRT-PCR.

All cDNA samples, standards and no-template controls were measured in triplicate in a 96-well plate covered with adhesive seals. For all measurements, 1 µL of cDNA template per 20 µL of final reaction volume was used on a StepOnePlus Real-time PCR System (Applied Biosystems) based on the SyGreen intercalating dye and a passive reference ROX (PCR Biosystems). All primers (Sigma-Aldrich; *SI Appendix*, Table S1) had a final concentration of 400 nM each. Reactions started with 3 min at 95 °C, followed by 40 cycles of 15 s at 95 °C and 30 s at primer melting temperature (Tm) (*SI Appendix*, Table S1). This reaction was followed by a melting curve, stepwise increasing temperature each 15 s by 0.5 °C, ranging from 65 °C to 95 °C. LinRegPCR (81) version 2016.1 was used for baseline correction (82), and quantification cycle values were loaded into qBase Plus version 3.2 (83) for relative quantity analysis. Amplification efficiencies were calculated for all primer sets and indicated 91 to 107% efficiency. Six genes were assessed for their suitability as reference genes using geNorm (84), identifying the optimal number of reference genes to use for gene expression normalization. The selected reference genes were used for further analysis.

### Immunostaining and Fluorescence Microscopy.

For cell characterization, cells were fixed in 3.7% vol/vol paraformaldehyde (Sigma-Aldrich) for 15 min, permeabilized with 0.2% vol/vol Triton X-100 (Sigma-Aldrich) for 10 min, and blocked with 3% vol/vol goat serum (Sigma-Aldrich) for 30 min. Cells were then incubated for 1 h with primary antibodies, NANOG (1:200; Invitrogen), OCT4 (1:200; Invitrogen), SOX2 (1:200; Invitrogen), Nestin (1:500; Millipore), PAX6 (1:200; Sigma-Aldrich), and βIII-tubulin (1:1,000; Sigma-Aldrich), followed with NucBlue Live ReadyProbes Reagent (Invitrogen) or Hoechst 33342 staining solution (Thermo Fisher Scientific) and Alexa Fluor secondary antibodies (Thermo Fisher Scientific) for 30 min. For iPSCs, Alexa Fluor 488 Phalloidin (Thermo Fisher Scientific) was also incubated with the nuclear stains and the Alexa Fluor secondary antibodies for visualizing cell outlines. Each step described above was followed by three washes with PBS. The stained samples were stored at 4 °C. Images for cell characterization were acquired with a Nikon Eclipse Ti-E inverted fluorescence microscope (Nikon Instruments, Inc.).

### MACS.

Expanded hiPSC-derived NSCs were dissociated using Accutase (STEMCELL Technologies) into single cells. The cells (∼1 × 10^7^ cells) were then tagged with Anti-PSA-NCAM MicroBeads (Miltenyi Biotec) for MACS according to the manufacturer's instructions. Briefly, the cells were blocked in MACS buffer [0.5% vol/vol BSA (Sigma-Aldrich) in PBS supplemented with 2 mM EDTA (Sigma-Aldrich)] for 10 min at 4 °C. The cells were then incubated with Anti-PSA-NCAM MicroBeads for 15 min at 4 °C. After extensive washing with MACS buffer, the cell suspension was loaded into a separation column (LS column) which was initially attached to a magnetic stand. Negatively labeled cells passed through the column after three washes with MACS buffer. Positively labeled cells which remained in the column were finally eluted to another tube with F20 Medium after removing the column from the magnetic stand.

### Measurements and Analysis of SCRS.

Cells were dissociated into single cells and fixed in 4% vol/vol paraformaldehyde for 15 min and were washed three times with PBS. Before Raman measurements, the fixed cells were dropped onto an aluminum-coated Raman substrate to be air dried. SCRS were acquired using an HR Evolution confocal Raman microscope (Horiba Jobin-Yvon) equipped with a 532-nm neodymium-yttrium aluminum garnet laser. The laser power on cells was 12 mW after attenuation by neutral density filters. An objective with a magnification of 50× was used to focus single cells with a laser spot size of ∼1 μm^2^, and Raman scattering was detected by a charge-coupled device cooled at −70 °C. The spectra were acquired in the range of 320 cm^−1^ to 3,400 cm^−1^ with a 300 grooves per mm diffraction grating. A mapping mode was used to characterize single cells pooled from three biological replicates, and the acquisition parameters were 5 s per spectrum, at least 20 spectra per cell, and 20 single cells per technical replicate, resulting in a total number of 8,774 SCRS from 536 cells (three biological replicates and 180 cells per biological replicate, 540 cells in total, but discarding the data from 4 cells due to the poor data quality). Each sample was performed in three biological replicates and three technical replicates. All SCRS were preprocessed by comic ray correction and polyline baseline fitting with LabSpec 6 (Horiba Scientific). Spectral normalization was done by vector normalization of the entire spectral region. The choice of vector normalization was made to correct general instrumentation fluctuation as well as sample and experimental variables (e.g., thickness of the sample) without strongly interfering with the nature of the biological content. Normalization using a particular biocomponent, such as nucleic acids or Amide I peak, was not used here, to avoid any presumptions of specific biomolecular changes. Data analysis, statistics, and visualization were done under an R 3.3.3 environment using in-house scripts (*SI Appendix*).

### t-SNE and Machine Learning Classification Models.

Due to the high dimensionality and collinearity of SCRS, t-SNE was used to embed the high-dimensional SCRS in a two-dimensional space by minimizing the Kullback−Leibler divergence between the two probability distributions in respective dimensional spaces (85). In this study, t-SNE was implemented by “Rt-SNE” in R 3.3.3, built based on 8,774 SCRS of the hiPSCs and neural derivatives derived from three hiPSC lines (SB-AD3-1, 010S-1, and 014S-10), including 3,316 SCRS of iPSCs, 2,342 SCRS of NSCs, and 3,116 SCRS of neurons.

Machine learning models were applied to classify the developmental stage of single cells with the use of their SCRS. Classification based on different cell lines was not performed, due to the little difference found among different donors. The dataset was split into a training set and a testing set with a ratio of 0.75:0.25 (6,581 and 2,193 SCRS, respectively). While the training set was used to train a classification model, the testing set was used to evaluate the model performance. Either raw SCRS or t-SNE−transformed SCRS were used as the inputs into various models, and the performances from the two approaches were compared. Tenfold cross-validation with five repetitions was used during model construction. First, single classifiers were employed, and, second, a stacked ensemble model was built upon the results from the single classifiers. The single classifiers include kNN (86), SGB (87), RF (88), and SVMs (89). The kNN is a nonparametric and simple algorithm based on calculating Euclidean distance and feature similarity. SGB is a boosting technique to achieve better classification results by combining several weak classifiers. RF is an ensemble-learning method combining several decision trees. SVMs use hyperplanes to separate the data in a high-dimensional space. Both linear SVM and SVM with RBF kernel were used in our study. After establishing and evaluating the classification models based on the five classifiers, five models were stacked together to build a two-layer machine learning model for a better classification result. The prediction outcomes of the five models (kNN, SGB, RF, linear SVM, and RBF-kernel SVM) from the first layer were used as features for the second layer, which used the SGB algorithm. All models were constructed in an R 3.3.3 environment.

Performance measures for all models were computed as sensitivity and specificity for each class as well as an overall accuracy rate.

Sensitivity=TP/(TP+FN)

Specificity=TN/(TN+FP)

Accuracy=(TP+TN)/(TP+TN+FP+FN),

where TP is the number of true positives, FP is the number of false positives, TN is the number of true negatives, and FN is the number of false negatives.

### Glycogen Assay.

The intracellular glycogen of cells was measured using the glycogen assay kit II (catalog no. ab169558; Abcam) according to the manufacturer’s instructions. Briefly, the cells (∼10^6^ cells per replicate) were washed with cold PBS and homogenized in 200 µL of ddH_2_O on ice. The homogenates were boiled for 10 min to inactivate enzymes in the sample, centrifuged for 10 min at 4 °C at 18,000 × *g* to remove any insoluble material, and stored at −80 °C for the glycogen assay. The absorbance was measured using a SpectraMax i3x Multi-Mode Microplate Reader System (Molecular Devices) at 450 nm.

### PAS Staining.

Fixed cells were incubated in 1% vol/vol periodic acid (Sigma-Aldrich) for 5 min, then stained with Schiff’s reagent (Sigma-Aldrich) for 15 min, followed by counterstaining with hematoxylin (Sigma-Aldrich) solution for 2 min. All steps were performed at room temperature, and the samples were rinsed with distilled water after each step. The samples were imaged under a microscope, and the cytoplasm of positive cells was stained purple-red.

### Statistical Analysis.

Data are presented as mean ± SEM, and sample size (n) indicates replicates from representative experiments. Experiments were performed with three biological replicates comprising at least three technical replicates, and one-way ANOVA with post hoc Tukey’s test was used throughout the study unless specified otherwise in the figure legends. A *P* value of <0.05 was considered statistically significant (in the diagrams, * represents *P* < 0.05, ** represents *P* ≤ 0.01, *** represents *P* ≤ 0.001, **** represents *P* ≤ 0.0001, and n.s. = not significant).

## Supplementary Material

Supplementary File

## Data Availability

Data and code have been made available in *SI Appendix* and on the Open Science Framework (https://osf.io/9env5/).
