# Supplementary material for: A single-cell Raman-based platform to identify developmental stages of human pluripotent stem cell-derived neurons
Source: Proc Natl Acad Sci U S A. 2020 Jul 21;117(31):18412–23. doi: 10.1073/pnas.2001906117 (PMC7414136; doi:10.1073/pnas.2001906117)
Supplement: Supplementary File [file pnas.2001906117.sapp.pdf]

PNAS
www.pnas.org

Supplementary Information for

A Single-Cell Raman-based Platform to Identify Developmental Stages of Human Pluripotent Stem Cell-derived Neurons

Chia-Chen Hsu[a, 1], Jiabao Xu[b, 1], Bas Brinkhof[a], Hui Wang[a], Zhanfeng Cui[a], Wei E. Huang[b]*, Hua Ye[a]*

[a]Institute of Biomedical Engineering, Old Road Campus Research Building, University of Oxford, Headington, Oxford OX3 7DQ, United Kingdom

[b]Department of Engineering Science, University of Oxford, Parks Road, Oxford OX1 3PJ, United Kingdom

[1]These authors contributed equally to this work.

*Corresponding authors. E-mail address: hua.ye@eng.ox.ac.uk (H. Ye); wei.huang@eng.ox.ac.uk (W. Huang).

This PDF file includes:

Figures S1 to S9

Tables S1 to S2

Materials and Methods

In-house scripts for Raman data analysis, statistics, and visualization

SI References

www.pnas.org/cgi/doi/10.1073/pnas.2001906117

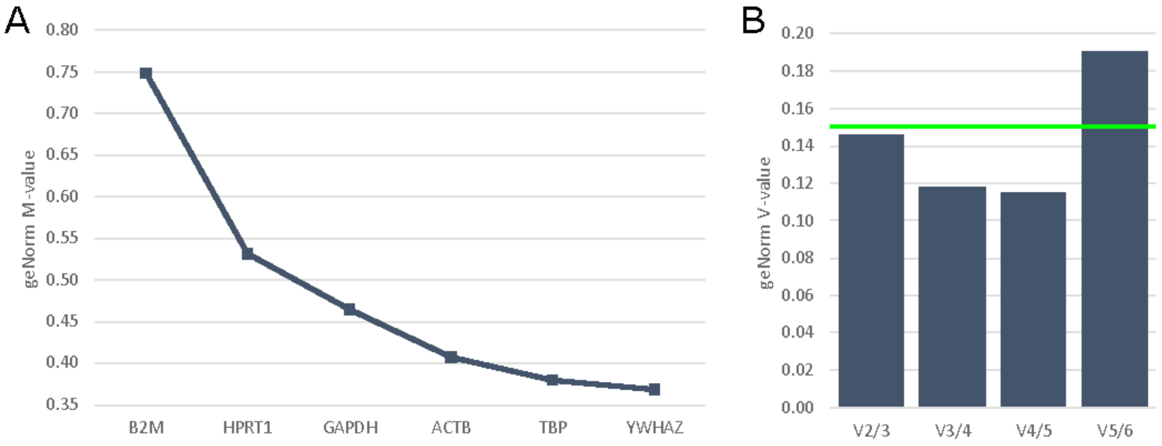

Figure S1. GeNorm analysis. (A) The reference target stability (M-value) for all genes was depicted and subsequently the least stable candidate reference gene was eliminated (X-Axis). (B) Pairwise variations (V-values) between the use of the two and three (V2/3), three and four (V3/4), four and five (V4/5) or five and six (V5/6) most stably expressed genes. The green line indicates the cut-off value set at 0.15. TBP and YWHAZ were selected as the reference genes for qPCR in this study.

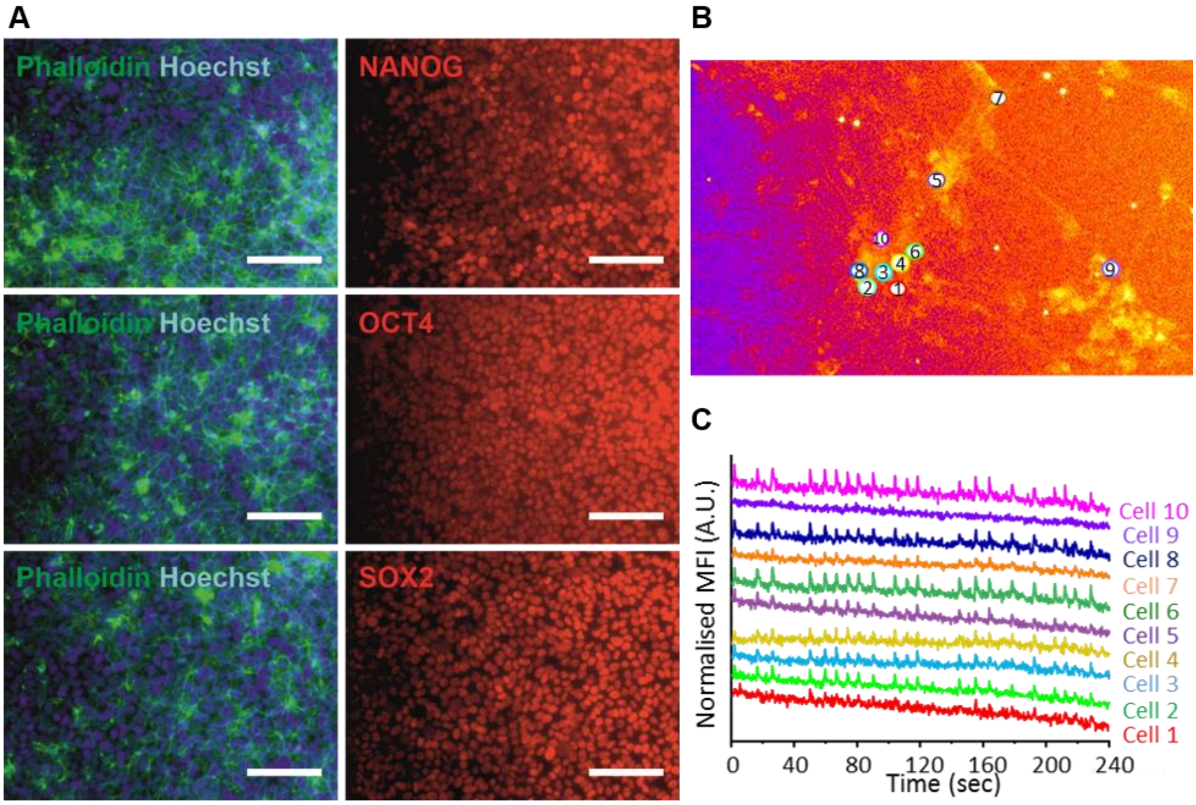

Figure S2. Protein and functional characterizations of the hiPSC-derived neural system. (A) Immunostaining was performed with different protein markers in hiPSCs (scale bars, 100 μm). (B–C) Ca^{2+} imaging revealed that after 7 weeks of differentiation, the selected cells (numbered in B) acquired Ca^{2+} transient (spikes; lines corresponding to the specific cells numbered in B), indicating they were capable of transducing Ca^{2+}-mediated signals in the neuron.

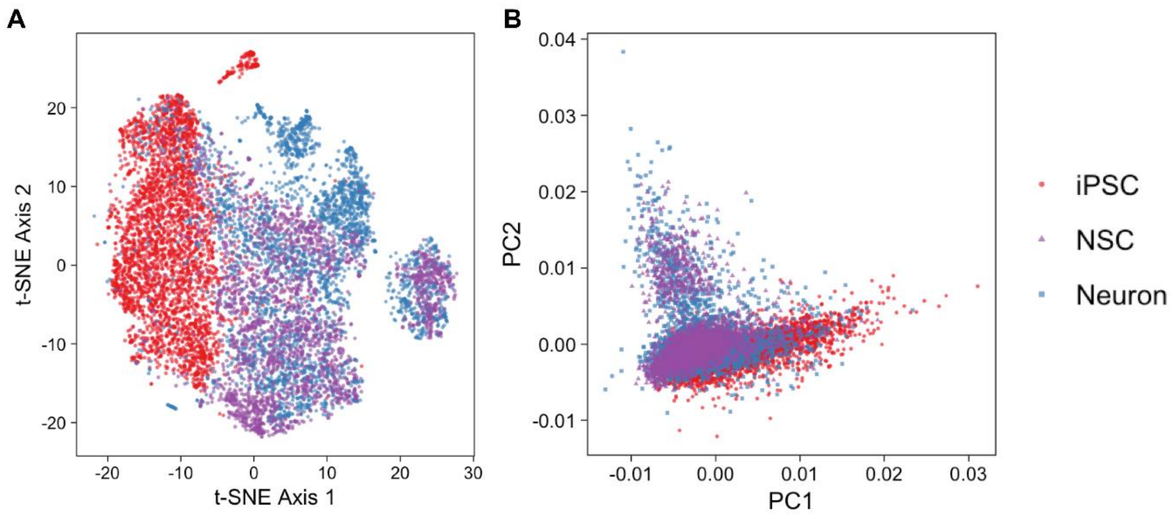

Figure S3. Comparison of the multivariate visualization. Multivariate visualization of the SCRS via (A) t-SNE and (B) PCA demonstrates a better separation between hiPSCs and their neural lineages using t-SNE (the red, purple, and blue colors represent iPSCs, NSCs, and neurons, respectively).

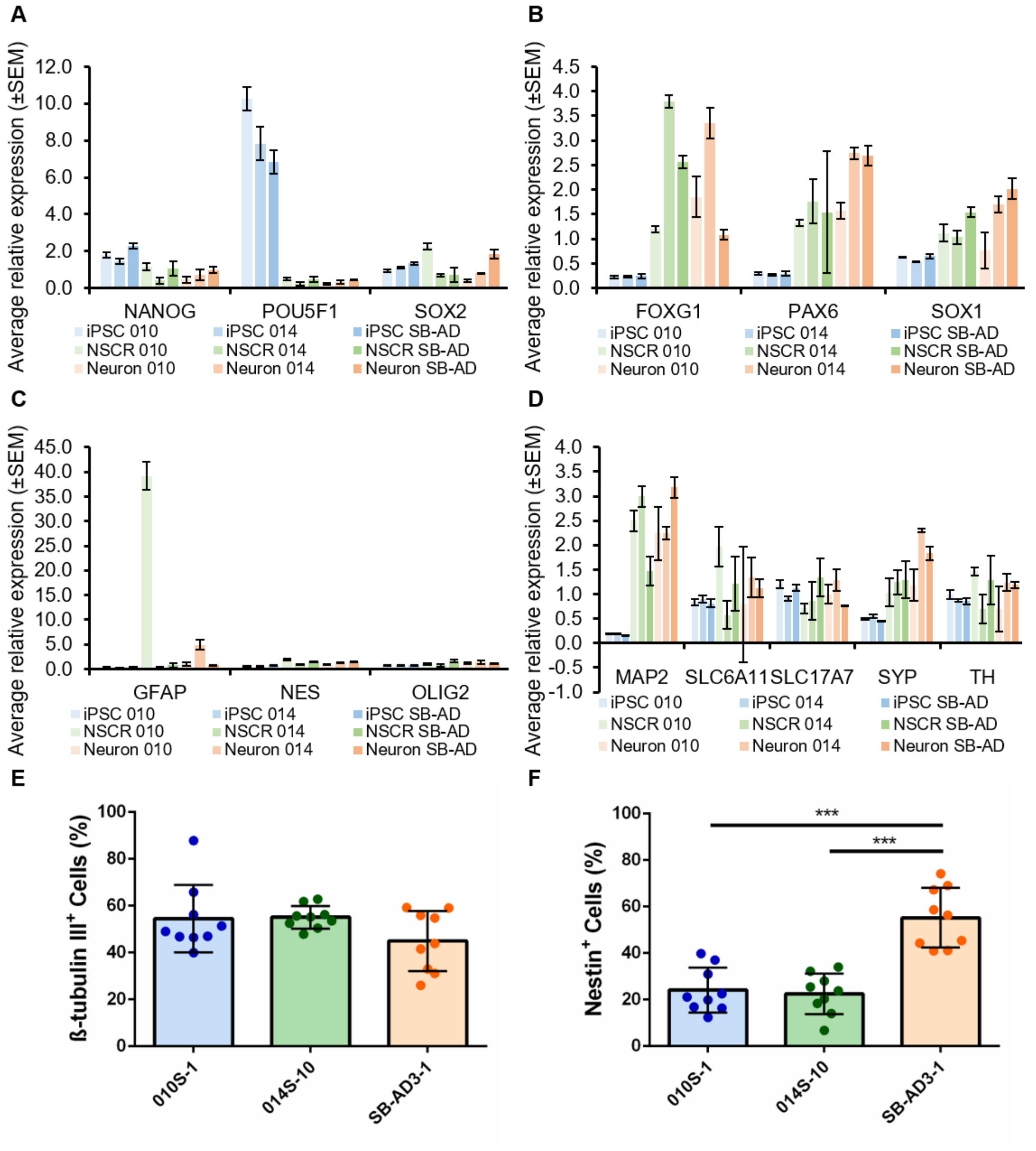

Figure S4. Comparisons of gene and protein expression levels between hiPSC-derived neural systems derived from different cell lines. Gene expression levels of cells from different cell lines and various developmental stages, including (A) Pluripotency genes (*NANOG*, *POU5F1*, and *SOX2*), (B) Neuroepithelial genes (*FOXG1*, *PAX6*, and *SOX1*), (C) Neural genes (*GFAP*, *NES*, and *OLIG2*), and (D) Neuronal genes (*SLC17A7*, *SLC6A11*, *TH*, *MAP2*, and *SYP*). (One-way ANOVA with post hoc Tukey's test was used. All experiments were performed with three technical replicates ($n = 3$). The results represent means ± s.e.m. Line 010S-1 is denoted as 010, line 014S-10 is denoted as 014, and line SB-AD3-1 is denoted as SB-AD in each figure legend; results of statistical analysis were detailed in Figures S2-S5). Neuronal differentiation and NSC proliferation were examined after 2 weeks differentiation and quantified with (E) Percentage of βIII-tubulin^{+} cells and (F) Percentage of Nestin^{+} cells, respectively. (One-way ANOVA with post hoc Tukey's test was used. All experiments were

performed with three technical replicates, three fields were examined for each replicate ($n = 9$). The results represent means ± s.e.m. *** represents $p \leq 0.001$ and there was no statistical significance between the other groups.)

A

**NANOG**	iPSC 014	iPSC SB	NSC 010	NSC 014	NSC SB	Neuron 010	Neuron 014	Neuron SB
iPSC 010	0.9942	0.9657	0.8394	0.0630	0.8818	0.0687	0.3222	0.5861
iPSC 014		0.5528	0.9990	0.3720	0.9997	0.3929	0.8467	0.9738
iPSC SB			0.1869	0.0024	0.2256	0.0026	0.0241	0.0725
NSC 010				0.7922	>0,9999	0.8109	0.9948	>0,9999
NSC 014					0.7373	>0,9999	0.9972	0.9545
NSC SB						0.7581	0.9895	0.9998
Neuron 010							0.9980	0.9617
Neuron 014								>0,9999

B

**POU5F1**	iPSC 014	iPSC SB	NSC 010	NSC 014	NSC SB	Neuron 010	Neuron 014	Neuron SB
iPSC 010	<0,0001	<0,0001	<0,0001	<0,0001	<0,0001	<0,0001	<0,0001	<0,0001
iPSC 014		0.2413	<0,0001	<0,0001	<0,0001	<0,0001	<0,0001	<0,0001
iPSC SB			<0,0001	<0,0001	<0,0001	<0,0001	<0,0001	<0,0001
NSC 010				0.9998	>0,9999	0.9994	>0,9999	>0,9999
NSC 014					0.9994	>0,9999	>0,9999	>0,9999
NSC SB						0.9986	>0,9999	>0,9999
Neuron 010							>0,9999	0.9999
Neuron 014								>0,9999

C

**SOX2**	iPSC 014	iPSC SB	NSC 010	NSC 014	NSC SB	Neuron 010	Neuron 014	Neuron SB
iPSC 010	>0,9999	0.9890	0.0607	0.9998	>0,9999	0.9312	>0,9999	0.4038
iPSC 014		0.9999	0.1714	0.9842	0.9996	0.7208	0.9972	0.6978
iPSC SB			0.4153	0.8526	0.9736	0.4006	0.9365	0.9327
NSC 010				0.0137	0.0422	0.0014	0.0261	0.9903
NSC 014					>0,9999	0.9980	>0,9999	0.1466
NSC SB						0.9643	>0,9999	0.3219
Neuron 010							0.9875	0.0235
Neuron 014								0.2332

Figure S5. Statistical analysis of qPCR for the pluripotent genes comparing three human iPSC lines. P-values calculated using the Two-way ANOVA with post hoc Tukey's test for the pluripotent genes, including (A) *NANOG*, (B) *POU5F1*, and (C) *SOX2*. ($p < 0.05$ is denoted in green, $p \leq 0.01$ is denoted in yellow, $p \leq 0.0001$ is denoted in orange, not significant is denoted in white.)

A

FOXG1	iPSC 014	iPSC SB	NSC 010	NSC 014	NSC SB	Neuron 010	Neuron 014	Neuron SB
iPSC 010	>0,9999	>0,9999	0.3586	<0,0001	<0,0001	0.0042	<0,0001	0.5163
iPSC 014		>0,9999	0.3596	<0,0001	<0,0001	0.0043	<0,0001	0.5175
iPSC SB			0.3949	<0,0001	<0,0001	0.0051	<0,0001	0.5571
NSC 010				<0,0001	0.0480	0.6895	0.0001	>0,9999
NSC 014					0.1103	0.0018	0.9854	<0,0001
NSC SB						0.8691	0.6060	0.0244
Neuron 010							0.0343	0.5234
Neuron 014								<0,0001

B

PAX6	iPSC 014	iPSC SB	NSC 010	NSC 014	NSC SB	Neuron 010	Neuron 014	Neuron SB
iPSC 010	>0,9999	>0,9999	0.2886	0.0121	0.0019	0.0755	<0,0001	<0,0001
iPSC 014		>0,9999	0.2445	0.0093	0.0014	0.0603	<0,0001	<0,0001
iPSC SB			0.2922	0.0124	0.0020	0.0768	<0,0001	<0,0001
NSC 010				0.9229	0.6127	0.9993	0.0363	0.0466
NSC 014					0.9996	0.9989	0.5235	0.5871
NSC SB						0.9352	0.8738	0.9104
Neuron 010							0.1655	0.2011
Neuron 014								>0,9999

C

SOX1	iPSC 014	iPSC SB	NSC 010	NSC 014	NSC SB	Neuron 010	Neuron 014	Neuron SB
iPSC 010	>0,9999	>0,9999	0.9470	0.9830	0.4261	0.9994	0.2154	0.0370
iPSC 014		>0,9999	0.8752	0.9457	0.3001	0.9950	0.1371	0.0204
iPSC SB			0.9610	0.9888	0.4668	0.9997	0.2435	0.0439
NSC 010				>0,9999	0.9875	0.9995	0.9066	0.4714
NSC 014					0.9579	>0,9999	0.8134	0.3419
NSC SB						0.8163	>0,9999	0.9624
Neuron 010							0.5713	0.1636
Neuron 014								0.9976

Figure S6. Statistical analysis of qPCR for the neuroepithelial genes comparing three human iPSC lines. P-values calculated using the Two-way ANOVA with post hoc Tukey's test for the neuroepithelial genes, including (A) *FOXG1*, (B) *PAX6*, and (C) *SOX1*. ($p < 0.05$ is denoted in green, $p \leq 0.01$ is denoted in yellow, $p \leq 0.001$ is denoted in blue, $p \leq 0.0001$ is denoted in orange, not significant is denoted in white.)

A

GFAP	iPSC 014	iPSC SB	NSC 010	NSC 014	NSC SB	Neuron 010	Neuron 014	Neuron SB
iPSC 010	>0,9999	>0,9999	<0,0001	>0,9999	0.9984	0.9893	<0,0001	>0,9999
iPSC 014		>0,9999	<0,0001	>0,9999	0.9979	0.9872	<0,0001	>0,9999
iPSC SB			<0,0001	>0,9999	0.9979	0.9871	<0,0001	>0,9999
NSC 010				<0,0001	<0,0001	<0,0001	<0,0001	<0,0001
NSC 014					0.9976	0.9858	<0,0001	0.9999
NSC SB						>0,9999	0.0004	>0,9999
Neuron 010							0.0009	>0,9999
Neuron 014								0.0002

B

NES	iPSC 014	iPSC SB	NSC 010	NSC 014	NSC SB	Neuron 010	Neuron 014	Neuron SB
iPSC 010	>0,9999	>0,9999	0.8102	>0,9999	0.9812	>0,9999	0.9958	0.9532
iPSC 014		>0,9999	0.7751	>0,9999	0.9727	>0,9999	0.9931	0.9374
iPSC SB			0.8867	>0,9999	0.9940	>0,9999	0.9992	0.9807
NSC 010				0.9570	0.9997	0.9437	0.9974	>0,9999
NSC 014					0.9993	>0,9999	>0,9999	0.9964
NSC SB						0.9988	>0,9999	>0,9999
Neuron 010							>0,9999	0.9943
Neuron 014								>0,9999

C

OLIG2	iPSC 014	iPSC SB	NSC 010	NSC 014	NSC SB	Neuron 010	Neuron 014	Neuron SB
iPSC 010	>0,9999	>0,9999	>0,9999	>0,9999	0.9819	0.9998	0.9983	>0,9999
iPSC 014		>0,9999	>0,9999	>0,9999	0.9709	0.9994	0.9963	>0,9999
iPSC SB			>0,9999	>0,9999	0.9790	0.9997	0.9978	>0,9999
NSC 010				>0,9999	0.9982	>0,9999	>0,9999	>0,9999
NSC 014					0.9924	>0,9999	0.9996	>0,9999
NSC SB						>0,9999	>0,9999	0.9991
Neuron 010							>0,9999	>0,9999
Neuron 014								>0,9999

Figure S7. Statistical analysis of qPCR for the neural genes comparing three human iPSC lines. P-values calculated using the Two-way ANOVA with post hoc Tukey's test for the neural genes, including (A) *GFAP*, (B) *NES*, and (C) *OLIG2*. ($p \leq 0.001$ is denoted in blue, $p \leq 0.0001$ is denoted in orange, not significant is denoted in white.)

A

MAP2	iPSC 014	iPSC SB	NSC 010	NSC 014	NSC SB	Neuron 010	Neuron 014	Neuron SB
iPSC 010	>0,9999	>0,9999	<0,0001	<0,0001	0.0612	<0,0001	0.0002	<0,0001
iPSC 014		>0,9999	<0,0001	<0,0001	0.0585	<0,0001	0.0002	<0,0001
iPSC SB			<0,0001	<0,0001	0.0462	<0,0001	0.0001	<0,0001
NSC 010				0.9650	0.3483	>0,9999	0.9995	0.8103
NSC 014					0.0227	0.8815	0.7096	>0,9999
NSC SB						0.5329	0.7457	0.0057
Neuron 010							>0,9999	0.6343
Neuron 014								0.4179

B

SLC17A7	iPSC 014	iPSC SB	NSC 010	NSC 014	NSC SB	Neuron 010	Neuron 014	Neuron SB
iPSC 010	0.9989	>0,9999	0.9683	>0,9999	0.9994	>0,9999	>0,9999	0.9812
iPSC 014		0.9999	>0,9999	>0,9999	0.9254	>0,9999	0.9884	>0,9999
iPSC SB			0.9890	>0,9999	0.9964	>0,9999	>0,9999	0.9944
NSC 010				0.9964	0.7068	0.9984	0.8907	>0,9999
NSC 014					0.9888	>0,9999	0.9995	0.9985
NSC SB						0.9803	>0,9999	0.7634
Neuron 010							0.9988	0.9994
Neuron 014								0.9232

C

SLC6A11	iPSC 014	iPSC SB	NSC 010	NSC 014	NSC SB	Neuron 010	Neuron 014	Neuron SB
iPSC 010	>0,9999	>0,9999	0.1265	>0,9999	0.8255	0.7898	0.8474	0.9987
iPSC 014		>0,9999	0.1750	>0,9999	0.8909	0.8628	0.9073	0.9998
iPSC SB			0.1093	>0,9999	0.7927	0.7542	0.8166	0.9976
NSC 010				0.0662	0.9412	0.9570	0.9289	0.4733
NSC 014					0.6705	0.6259	0.6993	0.9882
NSC SB						>0,9999	>0,9999	0.9947
Neuron 010							>0,9999	0.9912
Neuron 014								0.9963

D

SYP	iPSC 014	iPSC SB	NSC 010	NSC 014	NSC SB	Neuron 010	Neuron 014	Neuron SB
iPSC 010	>0,9999	>0,9999	0.8967	0.6416	0.4380	0.7106	0.0020	0.0570
iPSC 014		>0,9999	0.9319	0.7108	0.5084	0.7747	0.0029	0.0752
iPSC SB			0.8383	0.5503	0.3542	0.6221	0.0013	0.0394
NSC 010				>0,9999	0.9974	>0,9999	0.1310	0.7237
NSC 014					>0,9999	>0,9999	0.3354	0.9376
NSC SB						>0,9999	0.5283	0.9879
Neuron 010							0.2777	0.9044
Neuron 014								0.9781

E

TH	iPSC 014	iPSC SB	NSC 010	NSC 014	NSC SB	Neuron 010	Neuron 014	Neuron SB
iPSC 010	>0,9999	>0,9999	0.9745	>0,9999	0.9508	>0,9999	0.9995	>0,9999
iPSC 014		>0,9999	0.8928	>0,9999	0.8363	>0,9999	0.9911	0.9977
iPSC SB			0.8839	>0,9999	0.8251	>0,9999	0.9896	0.9972
NSC 010				0.8658	>0,9999	0.9203	>0,9999	0.9992
NSC 014					0.8026	>0,9999	0.9862	0.9959
NSC SB						0.8723	0.9995	0.9972
Neuron 010							0.9951	0.9989
Neuron 014								>0,9999

Figure S8. Statistical analysis of qPCR for the neuronal genes comparing three human iPSC lines. P-values calculated using the Two-way ANOVA with post hoc Tukey's test for the neuronal genes, including (A) *MAP2*, (B) *SLC17A7*, (C) *SLC6A11*, (D) *SYP*, and (E) *TH*.

($p < 0.05$ is denoted in green, $p \leq 0.01$ is denoted in yellow, $p \leq 0.001$ is denoted in blue, $p \leq 0.0001$ is denoted in orange, not significant is denoted in white.)

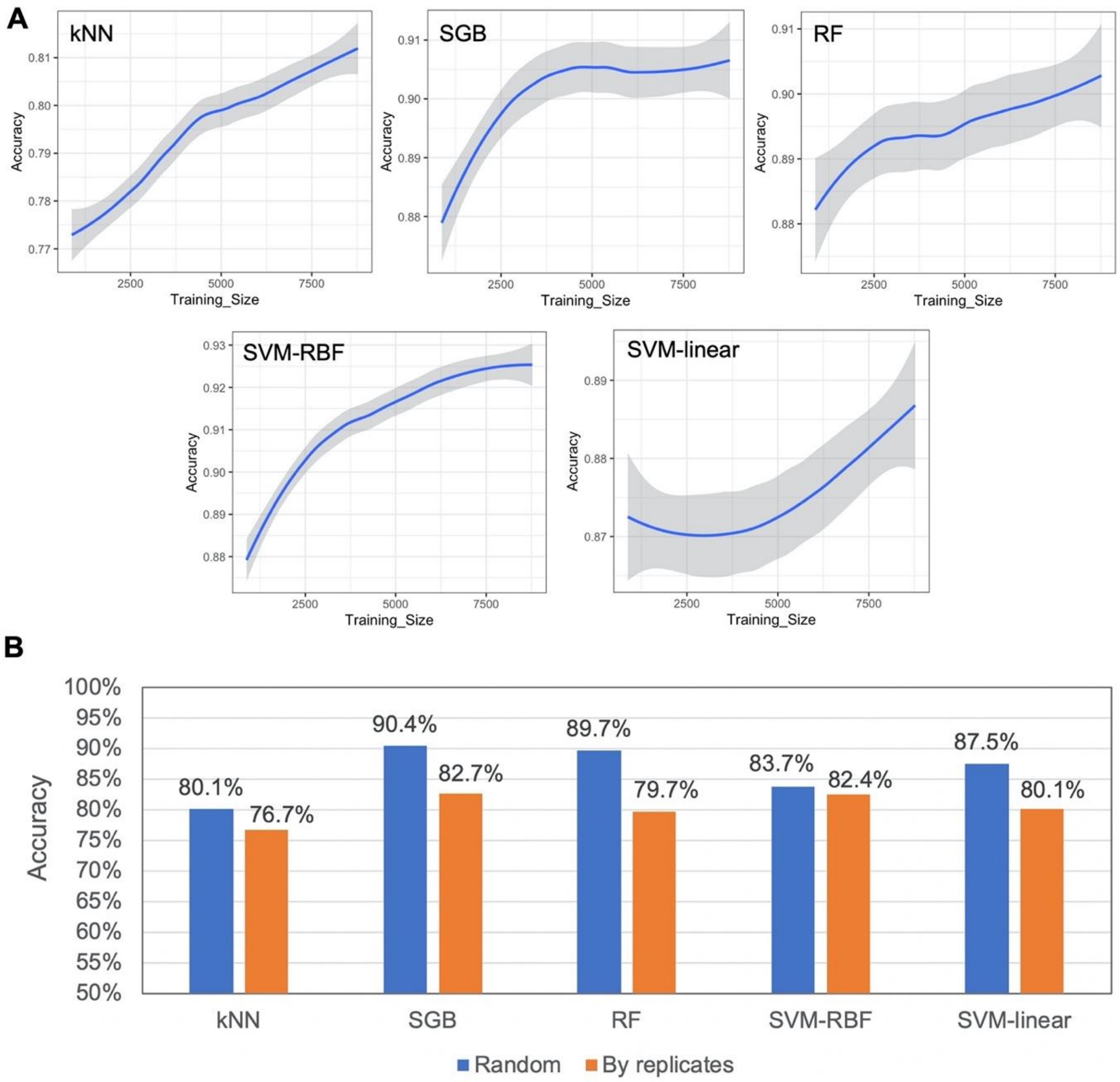

Figure S9. Learning curves of different machine learning models and the effect of the choice of different data splitting methods on the modeling performance. (A) Learning curves of five models were plotted as accuracy versus training set size. Four out of five models have decreased learning rate when the training size was around 5000. All models have good performance at the starting point with a training size of 10% of the entire dataset (n = 877), showing the effectiveness of our data size. (B) The effects of different data splitting methods by either random splitting or splitting by different biological replicates. The effects of using splitting by biological replicates were different across models, with the smallest decrease in SVM (1.3%) and the largest decrease in Random Forest (10%).

Table S1. Primer details for selected gene targets.

Gene symbol (Gene ID)	Full Gene Name (ref) (‡ noted for candidate reference gene)	NCBI ref seq	Primer sequence 5' --> 3'	Amplicon length (bp)	Ta (°C)
ACTB	actin beta‡ (a)	NM_001101	F-CACCAACTGGGACGACAT	189	60
(60)			R-ACAGCCTGGATAGCAACG		
B2M	beta-2-microglobulin‡ (a)	NM_004048	F-TAGCTGTGCTCGCGCT	224	62
(567)			R-AGACCAGTCCTTGCTGAAAGA		
FOXG1	forkhead box G1 (b)	NM_005249	F-CAAGGAGGGCGAGAAGAAGA	127	62
(2290)			R-GATGAACTCGTAGATGCCGTTG		
GAPDH	glyceraldehyde-3-phosphate dehydrogenase‡ (a)	NM_002046	F-GGATTTGGTCGTATTGGG	205	64
(2597)			R-GGAAGATGGTGATGGGATT		
GFAP	glial fibrillary acidic protein	NM_002055	F-CGCACGCAGTATGAGGCAA	179	62
(2670)			R-GACTCCAGGTCGCAGGTCAA		
HPRT1	hypoxanthine phosphoribosyltransferase 1‡ (c)	NM_000194	F-GACCAGTCAACAGGGGACAT	132	66
(3251)			R-CCTGACCAAGGAAAGCAAAG		
MAP2	microtubule associated protein 2 (d)	NM_002374	F-AACCGAGGAAGCATTGATTG	96	62
(4133)			R-TTCGTTGTGTCGTGTTCTCA		
NANOG	Nanog homeobox (e)	NM_024865	F-CCTCCAGCAGATGCAAGAACTC	169	59
(79923)			R-GTAAAGGCTGGGGTAGGTAGGTG		
NES	Nestin	NM_006617	F-ACCTGGCGGTGGCTC	131	62
(10763)			R-GTGAGGGGAGGGAAGTTGG		
OLIG2	oligodendrocyte transcription factor 2 (f)	NM_005806	F-CGACTCATCTTTCCTTCTCTAA	175	62
(10215)			R-CGCACTTACCTCATCATTG		
PAX6	paired box 6	NM_000280	F-CAGCAACAGGAAGGAGGG	174	62
(5080)			R-TGGATAATGGGTTCTCTCAAACT		
POU5F1	POU class 5 homeobox 1 (g)	NM_002701	F-AGCCCTCATTTCACCAGGCC	456	66
(5460)			R-TGGGACTCCTCCGGGTTTTG		
SLC17A7	solute carrier family 17 member 7 (h)	NM_020309	F-CCATGACTAAGCACAAGACTC	81	60
(57030)			R-AGATGACACCTCCATAGTGC		
SLC6A11	solute carrier family 6 member 11	NM_014229	F-CTGCGACATTCCCCTACATCA	144	62
(6538)			R-GCGTTCCAGCATCTACCCAG		
SOX1	SRY-box 1	NM_005986	F-GTGTCCCCCACTCACCTTCC	183	62
(6656)			R-GCCTCTCGCCTCGTTTTGAC		
SOX2	SRY-box 2 (i)	NM_003106	F-ACACCAATCCCATCCACACT	117	68
(6657)			R-CCTCCCCAGGTTTTCTCTGT		
SYP	synaptophysin (d)	NM_003179	F-AGGGAACACATGCAAGGAG	115	62
(6855)			R-CTTAAACACGAACCACAGG		
TBP	TATA-box binding protein‡ (a)	NM_003194	F-ATCAGAACAACAGCCTGCC	113	64
(6908)			R-GGTCAGTCCAGTGCCATAAG		
TH	tyrosine hydroxylase (h)	NM_199292	F-GTGCTAAACCTGCTCTTCTC	81	60
(7054)			R-TTCAAACGTCTCAAACACCT		
YWHAZ	tyrosine 3-monooxygenase/tryptophan 5-monooxygenase activation protein zeta‡ (a)	NM_145690	F-TCATCTTGGAGGGTCGTCT	180	64
(7534)			R-GACTTTGCTCTCTGCTTGTG		

Table S2. Classification of hiPSCs and hiPSC-derived neural lineage cells with various machine-learning classifiers (8774 spectra in total; train:test = 0.75:0.25; 6581 and 2193 spectra, respectively, cv = 10, repeats = 5).

	Model	Accuracy
Single classifier	kNN (k = 9)	78.9%
	Stochastic Gradient Boosting	91.0%
	Random Forest	88.6%
	SVM (RBF kernel, C = 1)	92.8%
	SVM (linear, C = 0.25)	92.8%
t-SNE + single classifier	kNN + t-SNE (k = 7)	82.3%
	GBM + t-SNE	91.0%
	Random Forest + t-SNE	90.0%
	SVM + t-SNE (RBF kernel, C = 1)	92.7%
	SVM + t-SNE (linear, C = 0.25)	88.5%
Stacked SGB		96.7%
t-SNE-transformed Stacked SGB		97.5%

Materials and Methods

Calcium Imaging for Neuronal Activity

Calcium activity was imaged using Oregon Green™ 488 BAPTA-2, AM calcium indicator (Thermo Fisher Scientific). Cells were incubated in a 4 μM dye solution in FluoroBrite DMEM imaging media (Thermo Fisher Scientific) for 30 min, washed twice in PBS, and further incubated in fresh imaging media for 30 min. Cells were imaged using a standard FITC/GFP filter at 10 fps continuously for 240 seconds. Random cells picked within the selected field were traced and the neuronal activity was evaluated by changes in the mean fluorescence intensity (MFI) of the cell over the background in each frame normalized to the average MFI throughout the recorded period.

Learning Curves of Machine Learning Models and the Effect of Different Data Splitting Methods on the Modeling Performance

Learning curves of the five models were built with training sizes from 10%, 20%, 30%, 40%, 50%, 60%, 70%, 80%, 90% to 100% and 25-time cross validation. Learning curves were plotted as accuracy versus training size to illustrate the effect of the size of the dataset on the model performance.

Generally, training/validation/testing sets containing data from the same distribution is preferred to optimize the model performance. Most previous studies using Raman spectroscopy in machine learning have utilized similar data-splitting method as well as leave-one-out cross-validation method (j–m). However, to achieve better clinical relevance, we have compared the effect of different data splitting methods by either random partitioning or partitioning by biological replicates. All five models were re-built using spectra from two biological replicates as the training set and one biological replicate as the validation set. The results were compared with those by random data splitting with the same training/validation size. As for the purpose

of this manuscript, we aim to train a model to its best performance for future applications. Therefore, a random data splitting was used as the main model training method to ensure that the model has utilized all possible resources.

In-house scripts for Raman data analysis, statistics, and visualization

```
# Set working directory to the folder containing spectra in .txt
setwd("working directory")
files <- list.files(path = "working directory")

# Create a dataframe contaning all spectra
combinedata <- read.table(files[1], header = FALSE, sep = "\t")[1,]
combinedata$Filename <- "Filename"
for (filename in files)
{
  data <- read.table(filename, header = FALSE, sep="\t")[-1,]
  data$Filename <- filename
  combinedata <- rbind(combinedata, data)
}
row.names(combinedata)[1] <- "Wavenumber"
combinedata <- combinedata[,-c(1:2)]

# Remove rows with 0s
combinedata[combinedata==0] <- NA
combinedata <- combinedata[complete.cases(combinedata),]


grep("Filename",combinedata) #1020

# Count no of spectra in each group
tablea<-table(grep("iPSC",combinedata$Filename))
tableb<-table(grep("NSC",combinedata$Filename))
tablec<-table(grep("Neuron",combinedata$Filename))

# Create a numeric matrix
spec <- as.matrix(combinedata[-1,-1020]) # Numeric matrix for intensities
wn <- as.numeric(combinedata[1,-1020]) # Numeric vector for wavenumber
t.spec <- t(spec) # Transpose
str(t.spec)
str(wn)

# Plot spectra
matplot(wn, t.spec, type="l") # See all
# Select FP region :1800cm-1
spec <- spec[,c(1:443)]
# t.spec <- t.spec[c(1:443),]
wn <- wn[c(1:443)]


############ Baseline correction
library(baseline)
spec.bl <- baseline(spec, method='rollingBall',wm=200, ws=100)

# Plot any one orginal vs corrected spectrum
plot(spec.bl, specNo = 1053)

# Output
corr <- getCorrected(spec.bl)
t.corr <- t(corr)

# Plot
```

```
matplot(wn, t.spec, type="l") # orginal spectra
matplot(wn, t.corr, type="l") # baseline-corrected spectra


############# Vector normalisation
# normalize a row by the sum of its entries
for (n in 1:nrow(corr)) {
  S <- sum(corr[n,]) # Add range here if specfic wn desired
  corr[n,] <- corr[n,]/S
}

t.corr <- t(corr)
# rownames(corr) <- wn
# colnames(t.corr) <- wn
# Plot
matplot(wn, t.corr, type="l")


# As data frame and change condition name
data <- data.frame(corr, row.names = NULL)
colnames(data) <- wn

# Add file name
library(stringr)
str(data)
data$File <- combinedata[-1,-(444:1019)]$Filename
data$Group <- word(data$File,1,sep = fixed("_"))
data$CellLine <- word(data$File,2,sep = fixed("_"))
data$Replicate <-word(data$File,3,sep = fixed("_"))
data$Time <-word(data$File,4,sep = fixed("_"))
data$Cell <-word(data$File,5,sep = fixed("_"))


# Find col index for class and variables
grep("File", colnames(data)) #444 #1020
grep("Group", colnames(data)) #445
grep("CellLine", colnames(data)) #446
grep("Replicate", colnames(data)) #447
grep("Time", colnames(data)) #448
grep("Cell", colnames(data)) #449


###### Plot spectra
library(reshape2)
library(plyr)
# Wide to long
data.long <- melt(data, variable.name = "Wavenumber",
              idvar = c("File","Group","CellLine", "Replicate", "Time", "Cell"))
data.long$Wavenumber <- as.numeric(as.character(data.long$Wavenumber))

# Summary stats
stat.1 <- ddply(data.long, c("Group", "CellLine","Wavenumber"), summarise,
             average = mean(value), stdev = sd(value),
             error = sd(value)/sqrt(length(value)))

stat.1$Group <- factor(stat.1$Group, levels = c("iPSC","NSC","Neuron"))
stat.1$CellLine <- as.factor(stat.1$CellLine)
```

```
stat.1$modified_average = stat.1$average - 0.005 * as.numeric(as.factor(stat.1$Group))

# Plot averages and ribbons
library(ggplot2)
library(RColorBrewer)
display.brewer.all()
mypalette = brewer.pal(8, "Set1")[c(1,4,2)]
mypalette2 = brewer.pal(8, "Set2")[c(1,2,3)]

# Plot according to diff cell stages
SpectraPlot <- ggplot(stat.1, aes(x = Wavenumber, y = modified_average, group = Group)) +
  geom_line(aes(color = Group),size=0.6) +
  geom_ribbon(aes(ymin = modified_average - stdev,
                  ymax = modified_average + stdev,
                  fill = Group),
              alpha = 0.5) +
  labs(title="",x=expression(Wavenumber/cm^{"-1"}), y = "Intensity (a.u.)") +
  scale_fill_manual(values = mypalette) +
  scale_color_manual(values = mypalette) +
  scale_x_continuous(breaks=seq(300,1900,300)) +
  # coord_cartesian(ylim = c(0, 5)) +
  theme_linedraw() +
  theme(panel.grid.major = element_blank(),
        panel.grid.minor = element_blank(),
        legend.position = "right",
        legend.background = element_blank(),
        legend.title = element_blank(),
        axis.text.y = element_blank(),
        axis.ticks.y = element_blank()
  ) + ggtitle("") +
  facet_grid(.~CellLine)


SpectraPlot


# Plot according to diff cell lines
stat.1$modified_average = stat.1$average - 0.005 * as.numeric(as.factor(stat.1$CellLine))

ggplot(stat.1, aes(x = Wavenumber, y = modified_average, group = CellLine)) +
  geom_line(aes(color = CellLine)) +
  geom_ribbon(aes(ymin = modified_average - stdev,
                  ymax = modified_average + stdev,
                  fill = CellLine),
              alpha = 0.5) +
  labs(title="",x=expression(Wavenumber/cm^{"-1"}), y = "Intensity (a.u.)") +
  scale_fill_manual(values = mypalette2) +
  scale_color_manual(values = mypalette2) +
  scale_x_continuous(breaks=seq(300,1900,200)) +
  # coord_cartesian(ylim = c(0, 5)) +
  theme_linedraw() +
  theme(panel.grid.major = element_blank(),
        panel.grid.minor = element_blank(),
        legend.position = "top",
        legend.background = element_blank(),
        legend.title = element_blank(),
```

```
      axis.text.y = element_blank(),
      axis.ticks.y = element_blank()
  ) + ggtitle("") +
  facet_grid(.~Group)



####### PCA
library(FactoMineR)
library(factoextra)
data$Group <- factor(data$Group, levels = c("iPSC","NSC","Neuron"))
pca <- PCA(data[, -(444:449)], scale.unit = FALSE, ncp = 10,
         graph = FALSE)
PCA$data

# Visualize eigenvalues (scree plot)
# Show the percentage of variances explained by each principal component
fviz_eig(pca)


# Graph of individuals. Individuals with a similar profile are grouped together.
PCA <- fviz_pca_ind(pca, axes = c(1,2),
                  #select.ind = list(cos2 = 0.2),
                  label="none",
                  habillage = as.factor(data$Group),
                  repel = TRUE,
                  palette = mypalette,
                  addEllipses=TRUE, ellipse.level=0) +
  theme_linedraw() +
  labs(x = "PC1 (16.7%)", y = "PC2 (9.7%)")+
  # coord_cartesian(xlim=c(-0.02,0.025),ylim = c(-0.02,0.02))+
  theme(panel.grid.major = element_blank(),
      panel.grid.minor = element_blank(),
      legend.position = "right",
      legend.title = element_blank()) +
  ggtitle("")

plot(PCA)

ggplot(PCA$data, aes(x, y)) +
  geom_point(aes(color = Groups, shape = Groups),size=0.5,alpha=0.7)+
  scale_fill_manual(values = mypalette) +
  scale_color_manual(values = mypalette) +
  # coord_cartesian(ylim = c(0, 5)) +
  theme_linedraw() +
  labs(x = "PC1 (16.7%)", y = "PC2 (9.7%)")+
  theme(panel.grid.major = element_blank(),
      panel.grid.minor = element_blank(),
      legend.position = "right",
      legend.background = element_blank(),
      legend.title = element_blank()
  ) + ggtitle("")

# Graph of variables. Positive correlated variables point to the same side of the plot.
# Negative correlated variables point to opposite sides of the graph
fviz_pca_var(pca,
           col.var = "contrib", # Color by contributions to the PC
```

```
        gradient.cols = c("White","Gray","Yellow","Orange","Red"),
        repel = TRUE     # Avoid text overlapping
)

# Results for PCA
library(factoextra)
# Eigenvalues
eig.val <- get_eigenvalue(pca)
eig.val

# Results for Variables
res.var <- get_pca_var(pca)
res.var$coord          # Coordinates
res.var$contrib        # Contributions to the PCs
res.var$cos2           # Quality of representation
# Results for individuals
res.ind <- get_pca_ind(pca)
res.ind$coord          # Coordinates
res.ind$contrib        # Contributions to the PCs
res.ind$cos2           # Quality of representation

######## LDA
library(MASS)
library(caret)
# Remove class label and other labels
dataLDA <- data[,-c(444,446:449)]
dataLDA$Group <- factor(dataLDA$Group, levels = c("iPSC","NSC","Neuron"))
# Build LDA model
lda <- lda(Group~., data = dataLDA)

# Proportion of trace explains the percentage of variances explained by each discriminant function
lda # LD1 - 77.6%, LD2 - 22.4%

# View loadings/slopes/coefficients/weights of each variable on each discriminant function
round(lda$scaling, 2)
loadings = data.frame(lda$scaling)
loadings$AbsVal_LD1 = (loadings$LD1^2)^0.5
loadings$AbsVal_LD2 = (loadings$LD2^2)^0.5
loadings$wn <- wn

# Use LDA to predict class in probability of assigning correctly
predictions <- predict(lda)
predictions
round(predictions$posterior, 2)

# A Stacked Histogram of the LDA Values
# Make a stacked histogram of the values of the discriminant function for the samples from different groups
ldahist(data = predictions$x[,1], g=dataLDA$Group) # 1st DF
ldahist(data = predictions$x[,2], g=dataLDA$Group) # 2nd DF

# Plot and visualise - Scatterplots of the Discriminant Functions
lda.data <- cbind(data, predict(lda)$x)
lda.data$Group <- factor(lda.data$Group, levels = c("iPSC","NSC","Neuron"))
ggplot(lda.data, aes(LD1, LD2)) +
  geom_point(aes(color = Group, shape = Group),size=0.5,alpha=0.7)+
  scale_fill_manual(values = mypalette) +
  scale_color_manual(values = mypalette) +
```

```
  # coord_cartesian(ylim = c(0, 5)) +
  theme_linedraw() +
  labs(x = "LD1 (77.6%)", y = "LD2 (22.4%)")+
  theme(panel.grid.major = element_blank(),
        panel.grid.minor = element_blank(),
        legend.position = "right",
        legend.background = element_blank(),
        legend.title = element_blank()
  ) + ggtitle("")

# Plot LDA loadings
ggplot(loadings, aes(x=wn, y=AbsVal_LD2)) +
  geom_line()+
  labs(title="",x=expression(Wavenumber/cm^{"-1"}), y = "Contribution to LD2") +
  scale_fill_manual(values = mypalette) +
  scale_color_manual(values = mypalette) +
  scale_x_continuous(breaks=seq(200,1800,100)) +
  # coord_cartesian(ylim = c(0, 5)) +
  theme_linedraw() +
  theme(panel.grid.major = element_blank(),
        panel.grid.minor = element_blank(),
        legend.position = "right",
        legend.background = element_blank(),
        legend.title = element_blank()
  ) + ggtitle("")



##### tSNE
library(Rtsne)
## Executing the algorithm on curated data
set.seed(329)
dataOrig <- data
data <- dataOrig[grep("iPSC", dataOrig$Group),]
data <- dataOrig[grep("NSC", dataOrig$Group),]
data <- dataOrig[grep("Neuron", dataOrig$Group),]

# 2D t-SNE
tsne <- Rtsne(data[,-c(444:449)], perplexity = 100,
              pca=T, pca_scale = T)
# 3D t-SNE
# tsne3D <- Rtsne(data[,-c(444:449)], perplexity = 100,
#                 pca=T, pca_scale = T, dims = 3)
#
# #display results of 3D t-SNE
# require(rgl)
# plot3d(tsne3D$Y,
#        col=c(rep(mypalette[1],3316),rep(mypalette[2],2342),rep(mypalette[3],3116)),
#        xlab = "x", ylab = "y", zlab = "z")
# legend3d("topright", legend = c("iPSC","NSC","Neuron"), pch = 16, col = mypalette)
# movie3d(spin3d(), duration=5, clean=FALSE, convert=FALSE, dir = "/Users/Jiabao/Desktop")

# ggplot
d_tsne <- data.frame(tsne$Y)

d_tsne$Group <- as.character(data$Group)
d_tsne$Group <- factor(d_tsne$Group, levels = c("iPSC","NSC","Neuron"))
```

```
count(d_tsne$Group)


d_tsne$CellLine <- as.factor(as.character(data$CellLine))
count(d_tsne$CellLine)

# display.brewer.all()



library(ggplot2)
# t-SNE colored according to diff groups/CellLines
library(RColorBrewer)
mypalette = brewer.pal(8, "Set1")[c(1,4,2)]

ggplot(d_tsne, aes(X1, X2, color = d_tsne$Group, shape=d_tsne$Group)) +
  geom_point(size=0.7,alpha=0.4)+
  labs(x="t-SNE Axis 1", y="t-SNE Axis 2") +
  scale_fill_manual(values = mypalette) +
  scale_color_manual(values = mypalette) +
  theme_linedraw() +
  theme(panel.grid.major = element_blank(),
        panel.grid.minor = element_blank(),
        legend.position = "right",
        # legend.background = element_blank(),
        legend.title = element_blank()
  ) + ggtitle("")

ggplot(d_tsne, aes(X1, X2, color = d_tsne$CellLine)) +
  geom_point(size=0.3,alpha=0.5)+
  labs(x="t-SNE Axis 1", y="t-SNE Axis 2") +
  scale_fill_manual(values = mypalette2) +
  scale_color_manual(values = mypalette2) +
  theme_linedraw() +
  theme(panel.grid.major = element_blank(),
        panel.grid.minor = element_blank(),
        legend.position = "right",
        # legend.background = element_blank(),
        legend.title = element_blank()
  ) + ggtitle("")



####quantification
###### Integration
library(plyr)
library(easyGgplot2)
library(ggpubr)
my_comparisons <- list( c("iPSC", "NSC"), c("iPSC", "Neuron"), c("NSC", "Neuron") )


d_tsne_raman$`1644-1682` <- rowSums(d_tsne_raman[,396:408])
d_tsne_raman$`843-864` <- rowSums(d_tsne_raman[,152:158])

# Cytochrome c
d_tsne_raman$`739-753` <- rowSums(d_tsne_raman[,122:126])
d_tsne_raman$`1114-1134` <- rowSums(d_tsne_raman[,232:238])
```

```
ggplot2.boxplot(d_tsne_raman,
            xName = "Group",
            yName = d_tsne_raman$`739-753`,
            groupName = "Group",
            backgroundColor = "white",
            ytitle = expression(bold(paste(Integral~of~Raman~band~at~"746"~cm^"-1"))),
            xtitle = "",
            addDot = T, dotSize = 0.5, dotPosition = "jitter",
            removePanelGrid = TRUE, PanelBorder = c(1, "solid", "black"),
            axisLine=c(0, "solid", "black"),
            legendPosition = "none",
            # xShowTickLabel = FALSE,
            outlier.shape=NA
)+
  coord_cartesian(ylim=c(0.003,0.029))+
  scale_fill_manual(values = mypalette) +
  scale_color_manual(values = mypalette) +
  stat_compare_means(label = "p.signif", method = "t.test",
                comparisons = my_comparisons,
                label.y = c(0.024,0.026,0.028))+
  theme(axis.text.x = element_text(size=11, face="bold"),
     axis.text.y=element_text(size=9, face="plain"),
     axis.title.y = element_text(size=12,face="bold",colour="#993333"))

ggplot2.boxplot(d_tsne_raman,
            xName = "Group",
            yName = d_tsne_raman$`1114-1134`,
            groupName = "Group",
            backgroundColor = "white",
            ytitle = expression(bold(paste(Integral~of~Raman~band~at~"1125"~cm^"-1"))),
            xtitle = "",
            addDot = T, dotSize = 0.5, dotPosition = "jitter",
            removePanelGrid = TRUE, PanelBorder = c(1, "solid", "black"),
            axisLine=c(0, "solid", "black"),
            legendPosition = "none",
            # xShowTickLabel = FALSE,
            outlier.shape=NA
)+
  coord_cartesian(ylim=c(0.00,0.042))+
  scale_fill_manual(values = mypalette) +
  scale_color_manual(values = mypalette) +
  stat_compare_means(label = "p.signif", method = "t.test",
                comparisons = my_comparisons,
                label.y = c(0.033,0.036,0.04))+
  theme(axis.text.x = element_text(size=11, face="bold"),
     axis.text.y=element_text(size=9, face="plain"),
     axis.title.y = element_text(size=12,face="bold",colour="#993333"))

# Carbohydrates
d_tsne_raman$`464-489` <- rowSums(d_tsne_raman[,44:51])
d_tsne_raman$`388-428` <- rowSums(d_tsne_raman[,23:34])

ggplot2.boxplot(d_tsne_raman,
            xName = "Group",
            yName = d_tsne_raman$`464-489`,
            groupName = "Group",
```

```
            backgroundColor = "white",
            ytitle = expression(bold(paste(Integral~of~Raman~band~at~"480"~cm^"-1"))),
            xtitle = "",
            addDot = T, dotSize = 0.5, dotPosition = "jitter",
            removePanelGrid = TRUE, PanelBorder = c(1, "solid", "black"),
            axisLine=c(0, "solid", "black"),
            legendPosition = "none",
            # xShowTickLabel = FALSE,
            outlier.shape=NA
)+
  coord_cartesian(ylim=c(0,0.04))+
  scale_fill_manual(values = mypalette) +
  scale_color_manual(values = mypalette) +
  stat_compare_means(label = "p.signif", method = "t.test",
                 comparisons = my_comparisons,
                 label.y = c(0.03,0.034,0.038))+
  theme(axis.text.x = element_text(size=11, face="bold"),
      axis.text.y=element_text(size=9, face="plain"),
      axis.title.y = element_text(size=12,face="bold",colour="#993333"))

ggplot2.boxplot(d_tsne_raman,
            xName = "Group",
            yName = d_tsne_raman$`388-428`,
            groupName = "Group",
            backgroundColor = "white",
            ytitle = expression(bold(paste(Integral~of~Raman~band~at~"400"~cm^"-1"))),
            xtitle = "",
            addDot = T, dotSize = 0.5, dotPosition = "jitter",
            removePanelGrid = TRUE, PanelBorder = c(1, "solid", "black"),
            axisLine=c(0, "solid", "black"),
            legendPosition = "none",
            # xShowTickLabel = FALSE,
            outlier.shape=NA
)+
  coord_cartesian(ylim=c(0,0.072))+
  scale_fill_manual(values = mypalette) +
  scale_color_manual(values = mypalette) +
  stat_compare_means(label = "p.signif", method = "t.test",
                 comparisons = my_comparisons,
                 label.y = c(0.06,0.064,0.07))+
  theme(axis.text.x = element_text(size=11, face="bold"),
      axis.text.y=element_text(size=9, face="plain"),
      axis.title.y = element_text(size=12,face="bold",colour="#993333"))

# DNA/RNA
d_tsne_raman$`715-732` <- rowSums(d_tsne_raman[,115:120])
d_tsne_raman$`771-788` <- rowSums(d_tsne_raman[,131:136])

ggplot2.boxplot(d_tsne_raman,
            xName = "Group",
            yName = d_tsne_raman$`715-732`,
            groupName = "Group",
            backgroundColor = "white",
            ytitle = expression(bold(paste(Integral~of~Raman~band~at~"726"~cm^"-1"))),
            xtitle = "",
            addDot = T, dotSize = 0.5, dotPosition = "jitter",
            removePanelGrid = TRUE, PanelBorder = c(1, "solid", "black"),
```

```
            axisLine=c(0, "solid", "black"),
            legendPosition = "none",
            # xShowTickLabel = FALSE,
            outlier.shape=NA
)+
  coord_cartesian(ylim=c(0,0.025))+
  scale_fill_manual(values = mypalette) +
  scale_color_manual(values = mypalette) +
  stat_compare_means(label = "p.signif", method = "t.test",
               comparisons = my_comparisons,
               label.y = c(0.02,0.0215,0.024))+
  theme(axis.text.x = element_text(size=11, face="bold"),
      axis.text.y=element_text(size=9, face="plain"),
      axis.title.y = element_text(size=12,face="bold",colour="#993333"))


ggplot2.boxplot(d_tsne_raman,
            xName = "Group",
            yName = d_tsne_raman$`771-788`,
            groupName = "Group",
            backgroundColor = "white",
            ytitle = expression(bold(paste(Integral~of~Raman~band~at~"780"~cm^"-1"))),
            xtitle = "",
            addDot = T, dotSize = 0.5, dotPosition = "jitter",
            removePanelGrid = TRUE, PanelBorder = c(1, "solid", "black"),
            axisLine=c(0, "solid", "black"),
            legendPosition = "none",
            # xShowTickLabel = FALSE,
            outlier.shape=NA
)+
  coord_cartesian(ylim=c(0,0.031))+
  scale_fill_manual(values = mypalette) +
  scale_color_manual(values = mypalette) +
  stat_compare_means(label = "p.signif", method = "t.test",
               comparisons = my_comparisons,
               label.y = c(0.025,0.027,0.03))+
  theme(axis.text.x = element_text(size=11, face="bold"),
      axis.text.y=element_text(size=9, face="plain"),
      axis.title.y = element_text(size=12,face="bold",colour="#993333"))


# Phe
d_tsne_raman$`993-1013` <- rowSums(d_tsne_raman[,196:202])
d_tsne_raman$`1024-1033` <- rowSums(d_tsne_raman[,205:206])

ggplot2.boxplot(d_tsne_raman,
            xName = "Group",
            yName = d_tsne_raman$`993-1013`,
            groupName = "Group",
            backgroundColor = "white",
            ytitle = expression(bold(paste(Integral~of~Raman~band~at~"1003"~cm^"-1"))),
            xtitle = "",
            addDot = T, dotSize = 0.5, dotPosition = "jitter",
            removePanelGrid = TRUE, PanelBorder = c(1, "solid", "black"),
            axisLine=c(0, "solid", "black"),
            legendPosition = "none",
            # xShowTickLabel = FALSE,
```

```
            outlier.shape=NA
)+
  coord_cartesian(ylim=c(0.01,0.1))+
  scale_fill_manual(values = mypalette) +
  scale_color_manual(values = mypalette) +
  stat_compare_means(label = "p.signif", method = "t.test",
                 comparisons = my_comparisons,
                 label.y = c(0.08,0.086,0.095))+
  theme(axis.text.x = element_text(size=11, face="bold"),
       axis.text.y=element_text(size=9, face="plain"),
       axis.title.y = element_text(size=12,face="bold",colour="#993333"))


ggplot2.boxplot(d_tsne_raman,
            xName = "Group",
            yName = d_tsne_raman$`1024-1033`,
            groupName = "Group",
            backgroundColor = "white",
            ytitle = expression(bold(paste(Integral~of~Raman~band~at~"1030"~cm^"-1"))),
            xtitle = "",
            addDot = T, dotSize = 0.5, dotPosition = "jitter",
            removePanelGrid = TRUE, PanelBorder = c(1, "solid", "black"),
            axisLine=c(0, "solid", "black"),
            legendPosition = "none",
            # xShowTickLabel = FALSE,
            outlier.shape=NA
)+
  coord_cartesian(ylim=c(0,0.01))+
  scale_fill_manual(values = mypalette) +
  scale_color_manual(values = mypalette) +
  stat_compare_means(label = "p.signif", method = "t.test",
                 comparisons = my_comparisons,
                 label.y = c(0.008,0.0087,0.0096))+
  theme(axis.text.x = element_text(size=11, face="bold"),
       axis.text.y=element_text(size=9, face="plain"),
       axis.title.y = element_text(size=12,face="bold",colour="#993333"))

# Proteins amides
d_tsne_raman$`1625-1694` <- rowSums(d_tsne_raman[,390:412])
d_tsne_raman$`1213-1282` <- rowSums(d_tsne_raman[,262:283])

ggplot2.boxplot(d_tsne_raman,
            xName = "Group",
            yName = d_tsne_raman$`1625-1694`,
            groupName = "Group",
            backgroundColor = "white",
            ytitle = expression(bold(paste(Integral~of~Raman~band~at~"1660"~cm^"-1"))),
            xtitle = "",
            addDot = T, dotSize = 0.5, dotPosition = "jitter",
            removePanelGrid = TRUE, PanelBorder = c(1, "solid", "black"),
            axisLine=c(0, "solid", "black"),
            legendPosition = "none",
            # xShowTickLabel = FALSE,
            outlier.shape=NA
)+
  coord_cartesian(ylim=c(0.03,0.2))+
  scale_fill_manual(values = mypalette) +
```

```
  scale_color_manual(values = mypalette) +
  stat_compare_means(label = "p.signif", method = "t.test",
                  comparisons = my_comparisons,
                  label.y = c(0.15,0.17,0.192))+
  theme(axis.text.x = element_text(size=11, face="bold"),
        axis.text.y=element_text(size=9, face="plain"),
        axis.title.y = element_text(size=12,face="bold",colour="#993333"))

ggplot2.boxplot(d_tsne_raman,
               xName = "Group",
               yName = d_tsne_raman$`1213-1282`,
               groupName = "Group",
               backgroundColor = "white",
               ytitle = expression(bold(paste(Integral~of~Raman~band~at~"1250"~cm^"-1"))),
               xtitle = "",
               addDot = T, dotSize = 0.5, dotPosition = "jitter",
               removePanelGrid = TRUE, PanelBorder = c(1, "solid", "black"),
               axisLine=c(0, "solid", "black"),
               legendPosition = "none",
               # xShowTickLabel = FALSE,
               outlier.shape=NA
)+
  coord_cartesian(ylim=c(0.03,0.13))+
  scale_fill_manual(values = mypalette) +
  scale_color_manual(values = mypalette) +
  stat_compare_means(label = "p.signif", method = "t.test",
                  comparisons = my_comparisons,
                  label.y = c(0.11,0.117,0.128))+
  theme(axis.text.x = element_text(size=11, face="bold"),
        axis.text.y=element_text(size=9, face="plain"),
        axis.title.y = element_text(size=12,face="bold",colour="#993333"))

# Lipids
d_tsne_raman$`1425-1473` <- rowSums(d_tsne_raman[,327:342])
d_tsne_raman$`1262-1272` <- rowSums(d_tsne_raman[,277:280])
d_tsne_raman$`1265/1440` <- d_tsne_raman$`1262-1272` / d_tsne_raman$`1425-1473`

ggplot2.boxplot(d_tsne_raman,
               xName = "Group",
               yName = d_tsne_raman$`1425-1473`,
               groupName = "Group",
               backgroundColor = "white",
               ytitle = expression(bold(paste(Integral~of~Raman~band~at~"1440"~cm^"-1"))),
               xtitle = "",
               addDot = T, dotSize = 0.5, dotPosition = "jitter",
               removePanelGrid = TRUE, PanelBorder = c(1, "solid", "black"),
               axisLine=c(0, "solid", "black"),
               legendPosition = "none",
               # xShowTickLabel = FALSE,
               outlier.shape=NA
)+
  coord_cartesian(ylim=c(0.03,0.14))+
  scale_fill_manual(values = mypalette) +
  scale_color_manual(values = mypalette) +
  stat_compare_means(label = "p.signif", method = "t.test",
                  comparisons = my_comparisons,
                  label.y = c(0.12,0.127,0.137))+
```

```
  theme(axis.text.x = element_text(size=11, face="bold"),
        axis.text.y=element_text(size=9, face="plain"),
        axis.title.y = element_text(size=12,face="bold",colour="#993333"))


ggplot2.boxplot(d_tsne_raman,
                xName = "Group",
                yName = d_tsne_raman$`1262-1272`,
                groupName = "Group",
                backgroundColor = "white",
                ytitle = expression(bold(paste(Integral~of~Raman~band~at~"1265"~cm^"-1"))),
                xtitle = "",
                addDot = T, dotSize = 0.5, dotPosition = "jitter",
                removePanelGrid = TRUE, PanelBorder = c(1, "solid", "black"),
                axisLine=c(0, "solid", "black"),
                legendPosition = "none",
                # xShowTickLabel = FALSE,
                outlier.shape=NA
)+
  coord_cartesian(ylim=c(0.005,0.025))+
  scale_fill_manual(values = mypalette) +
  scale_color_manual(values = mypalette) +
  stat_compare_means(label = "p.signif", method = "t.test",
                     comparisons = my_comparisons,
                     label.y = c(0.0205,0.022,0.024))+
  theme(axis.text.x = element_text(size=11, face="bold"),
        axis.text.y=element_text(size=9, face="plain"),
        axis.title.y = element_text(size=12,face="bold",colour="#993333"))

ggplot2.boxplot(d_tsne_raman,
                xName = "Group",
                yName = d_tsne_raman$`1265/1440`,
                groupName = "Group",
                backgroundColor = "white",
                ytitle = expression(bold(paste(Ratio~of~"1265/1440"~cm^"-1"))),
                xtitle = "",
                addDot = T, dotSize = 0.5, dotPosition = "jitter",
                removePanelGrid = TRUE, PanelBorder = c(1, "solid", "black"),
                axisLine=c(0, "solid", "black"),
                legendPosition = "none",
                # xShowTickLabel = FALSE,
                outlier.shape=NA
)+
  coord_cartesian(ylim=c(0.05,0.4))+
  scale_fill_manual(values = mypalette) +
  scale_color_manual(values = mypalette) +
  stat_compare_means(label = "p.signif", method = "t.test",
                     comparisons = my_comparisons,
                     label.y = c(0.32,0.35,0.385))+
  theme(axis.text.x = element_text(size=11, face="bold"),
        axis.text.y=element_text(size=9, face="plain"),
        axis.title.y = element_text(size=12,face="bold",colour="#993333"))


ggplot2.boxplot(d_tsne_raman,
                xName = "Group",
                yName = d_tsne_raman$`843-864`,
```

```
             groupName = "Group",
             backgroundColor = "white",
             ytitle = "Integral of Raman band",
             xtitle = "",
             addDot = T, dotSize = 0.5, dotPosition = "jitter",
             removePanelGrid = TRUE, PanelBorder = c(1, "solid", "black"),
             axisLine=c(0, "solid", "black"),
             legendPosition = "none",
             # xShowTickLabel = FALSE,
             outlier.shape=NA
)+
  coord_cartesian(ylim=c(0.005,0.04))+
  scale_fill_manual(values = mypalette) +
  scale_color_manual(values = mypalette) +
  stat_compare_means(label = "p.signif", method = "t.test",
                 comparisons = my_comparisons,
                 label.y = c(0.03,0.034,0.038))+
  theme(axis.ticks.x = element_blank(),
       axis.text.y=element_text(size=10, face="plain"),
       axis.title.y = element_text(size=14,face="bold",colour="#993333"))


ggplot2.boxplot(d_tsne_raman,
             xName = "Group",
             yName = d_tsne_raman$`993-1013`,
             groupName = "Group",
             backgroundColor = "white",
             ytitle = "Integral of Raman band",
             xtitle = "",
             addDot = T, dotSize = 0.5, dotPosition = "jitter",
             removePanelGrid = TRUE, PanelBorder = c(1, "solid", "black"),
             axisLine=c(0, "solid", "black"),
             legendPosition = "none",
             # xShowTickLabel = FALSE,
             outlier.shape=NA
)+
  coord_cartesian(ylim=c(0.01,0.11))+
  scale_fill_manual(values = mypalette) +
  scale_color_manual(values = mypalette) +
  stat_compare_means(label = "p.signif", method = "t.test",
                 comparisons = my_comparisons,
                 label.y = c(0.085,0.095,0.105))+
  theme(axis.ticks.x = element_blank(),
       axis.text.y=element_text(size=10, face="plain"),
       axis.title.y = element_text(size=14,face="bold",colour="#993333"))



########### ML
library(caret)
# Grep the indexes of labels
grep("File", colnames(data)) #444
grep("Group", colnames(data)) #445
grep("CellLine", colnames(data)) #446
grep("Replicate", colnames(data)) #447
grep("Time", colnames(data)) #448
grep("Cell", colnames(data)) #449
```

```
data$Group <- as.factor(data$Group)
data$CellLine <- as.factor(data$CellLine)
data$Replicate <- as.factor(data$Replicate)
data$Time <- as.factor(data$Time)
data$Cell <- as.factor(data$Cell)


# Create balanced splits of the data
set.seed(329)
trainIndex <- createDataPartition(data$Group, p = .8,
                                  list = FALSE,
                                  times = 1)
head(trainIndex)
dataTrain <- data[ trainIndex,]
dataTest  <- data[-trainIndex,]

# Train with tSNE
all <- cbind(data, tsne$Y) # or tsne3D$Y if adding three colunms
dataTrain2 <- all[ trainIndex,]
dataTest2  <- all[-trainIndex,]



############ Feature selection
# NO.1 Find highly correlated variables
library(mlbench)
library(caret)
correlationMatrix <- cor(dataTrain[,-c(444:449)])
print(correlationMatrix)
# find attributes that are highly corrected (ideally >0.75)
highlyCorrelated <- findCorrelation(correlationMatrix, cutoff=0.5)
# print indexes of highly correlated attributes
print(highlyCorrelated)
dataTrain[highlyCorrelated]

# NO.2 Variable importance
knnImp <- varImp(knnFit)
knn_varimp <- data.frame(varImp(knnFit, scale = FALSE)$importance)
svm_varimp <- data.frame(varImp(svmFitLin, scale = FALSE)$importance)
svm_varimp2 <- data.frame(varImp(svmFitRBF, scale = FALSE)$importance)
roc_imp$importance
gbmImp = varImp(gbmFit,numTrees = NULL,scale = FALSE)
plot(varImp(svmFitRBF),top = 50)
?varImp
write.csv(knn_varimp, file = "knn_varimp.csv")
write.csv(svm_varimp, file = "svm_rbf_varimp.csv")
write.csv(svm_varimp2, file = "svm_linear_varimp.csv")

knn_varimp$wn <- as.numeric(row.names(knn_varimp))
svm_varimp$wn <- as.numeric(row.names(svm_varimp))
svm_varimp2$wn <- as.numeric(row.names(svm_varimp2))

library(reshape2)
svm_varimp$NSC <- svm_varimp$NSC + 0.5
svm_varimp$iPSC <- svm_varimp$iPSC + 1
svm_varimp_long <- melt(svm_varimp, id.vars = "wn", value.name = "int",
```

```
                variable.name = "group")

svm_varimp_long$group <- factor(svm_varimp_long$group, levels = c("iPSC","NSC","Neuron"))

ggplot(svm_varimp_long, aes(x=wn, y=int,color=group,group=group)) +
  geom_line()+
  labs(title="",x=expression(Wavenumber/cm^{"-1"}), y = "Contribution to model") +
  scale_fill_manual(values = mypalette) +
  scale_color_manual(values = mypalette) +
  scale_x_continuous(breaks=seq(200,1800,100)) +
  # coord_cartesian(ylim = c(0, 5)) +
  theme_linedraw() +
  theme(panel.grid.major = element_blank(),
        panel.grid.minor = element_blank(),
        legend.position = "right",
        legend.background = element_blank(),
        legend.title = element_blank(),
        axis.text.y = element_blank(),
        axis.ticks.y = element_blank()
  ) + ggtitle("")



# NO.3 Feature selection by filtered random forest
filterCtrl <- sbfControl(functions = rfSBF, method = "repeatedcv", repeats = 5)
rfWithFilter <- sbf(dataTrain[,-c(444:449)], dataTrain[,-c(444:449)], sbfControl = filterCtrl)
rfWithFilter
predictors(rfWithFilter)

# NO.4 Feature selection by Recursive Feature Elimination
control <- rfeControl(functions=rfFuncs, method="cv", number=10)
results <- rfe(dataTrain[,-444], dataTrain[,444],
             sizes=c(1:8), rfeControl=control)

print(results)
predictors(results)
plot(results, type=c("g", "o"))



####### ML models
# Parameter tuning
fitControl <- trainControl(## 10-fold CV
  method = "repeatedcv",
  number = 10,
  ## repeated 5 times
  repeats = 5,
  allowParallel=TRUE)

fitControl2 <- trainControl(## 10-fold CV
  method = "repeatedcv",
  number = 10,
  ## repeated 5 times
  repeats = 5,
  allowParallel=TRUE,
  search = "random") # !!!! take extremely long
```

```
############ Diff models
library(doParallel)
library(tictoc)

# kNN - k best at 90
tic()
cl <- makeCluster(detectCores())
registerDoParallel(cl)
knnGrid <- expand.grid(k = c(5,10,20,30,40,50,60,70,80,90,100))
knnFit <- train(Group ~ ., data = dataTrain[,-c(444,446:449)],
             method = "knn",
             trControl = fitControl,
             tuneGrid = knnGrid)
stopCluster(cl)
registerDoSEQ()
toc()


# LDA - no need for grid expansion, dim = 2
tic()
cl <- makeCluster(detectCores())
registerDoParallel(cl)
ldaFit <- train(Group ~ ., data = dataTrain[,-c(444,446:449)],
             method = "lda2",
             trControl = fitControl)
stopCluster(cl)
toc()

# pls
tic()
plsGrid <- expand.grid(ncomp = c(1,3,5,7,9,11,13,15,17,19))
cl <- makeCluster(detectCores())
registerDoParallel(cl)
plsFit <- train(Group ~ ., data = dataTrain[,-c(444,446:449)],
             method = "pls",
             trControl = fitControl,
             tuneGrid = plsGrid)
stopCluster(cl)
toc()


# Random Forest
tic()
cl <- makeCluster(detectCores())
registerDoParallel(cl)
rfFit <- train(Group ~ ., data = dataTrain[,-c(444,446:449)],
            method = "ranger",
            trControl = fitControl)
stopCluster(cl)
toc()


# gbm
tic()
cl <- makeCluster(detectCores())
registerDoParallel(cl)
gbmGrid2 <-  expand.grid(interaction.depth = c(1,5,9,11,15),
```

```
                    n.trees = c(500,1000,1500,2000,2500,3000,3500,4000,4500,5000,5500),
                    shrinkage = 0.1,
                    n.minobsinnode = 10)
gbmFit2 <- train(Group ~ ., data = dataTrain[,-c(444,446:449)],
             method = "gbm",
             trControl = fitControl,
             tuneGrid = gbmGrid2,
             verbose = FALSE)
stopCluster(cl)
toc()


# svmlinear
tic()
cl <- makeCluster(detectCores())
registerDoParallel(cl)
svmFitLin2 <- train(Group ~ ., data = dataTrain2[,-c(444,446:449)],
                method = "svmLinear2", # or svmLinear
                trControl = fitControl)
stopCluster(cl)
toc()


# svmRBF
tic()
cl <- makeCluster(detectCores())
registerDoParallel(cl)
svmFitRBF <- train(Group ~ ., data = dataTrain[,-c(444,446:449)],
               method = "svmRadialSigma", # or svmRadialWeights
               trControl = fitControl)
stopCluster(cl)
toc()


# xgboost
tic()
cl <- makeCluster(detectCores())
registerDoParallel(cl)
xgbFit <- train(Group ~ ., data = dataTrain[,-c(444,446:449)],
            method = "xgbDART",
            trControl = fitControl)
stopCluster(cl)
registerDoSEQ()
toc()

# ANN
library(keras)
# install_keras(tensorflow = "gpu")
keras::unsearlize_model(object$finalModel$object)
tic()
cl <- makeCluster(detectCores())
registerDoParallel(cl)
annFit <- train(Group ~ ., data = dataTrain[,-c(444,446:449)],
            method = "mlpML",
            trControl = fitControl)
stopCluster(cl)
registerDoSEQ()
```

```
toc()


# Plotting the Resampling Profile
trellis.par.set(caretTheme())
plot(gbmFit)
plot(gbmFit2, metric = "Kappa", plotType = "level",
    scales = list(x = list(rot = 90)))
ggplot(gbmFit)

save(dataTrain, dataTest, file = "data.RData")
saveRDS(knnFit, "knnFit.rds")
saveRDS(gbmFit, "gbmFit.rds")
saveRDS(gbmFit2, "gbmFit2.rds")
saveRDS(plsFit, "plsFit.rds")
saveRDS(ldaFit, "ldaFit.rds")
saveRDS(svmFitLin, "svmFitLin.rds")
saveRDS(svmFitRBF, "svmFitRBF.rds")
saveRDS(rfFit, "rfFit.rds")
saveRDS(xgbFit, "xgbFit.rds")
knnFit <- readRDS("knnFit.rds")
ldaFit <- readRDS("ldaFit.rds")
gbmFit <- readRDS("gbmFit.rds")
gbmFit2 <- readRDS("gbmFit2.rds")
plsFit <- readRDS("plsFit.rds")
svmFitLin <- readRDS("svmFitLin.rds")
svmFitRBF <- readRDS("svmFitRBF.rds")

xgbFit$bestTune

xgbFit$results[416,]


# Compare models by resampling
resamps <- resamples(list(KNN = knnFit,
                    LDC = ldaFit,
                    PLS = plsFit,
                    RF = rfFit,
                    GBM = gbmFit,
                    XGB = xgbFit,
                    SVMlin = svmFitLin,
                    SVMRBF = svmFitRBF))
resamps
summary(resamps)
modelCor(resamps) # Models being stacked should have low correlations (<0.75)
splom(resamps)

# Save plots
jpeg("modelcorr.jpg", width = 4000, height = 4000, res = 300)
splom(resamps)
dev.off()

# Predict
knnPred <- predict(knnFit, dataTest[,-c(444:449)])
ldaPred <- predict(ldaFit, dataTest[,-c(444:449)])
plsPred <- predict(plsFit, dataTest[,-c(444:449)])
rfPred <- predict(rfFit, dataTest[,-(444:449)])
```

```
gbmPred <- predict(gbmFit, dataTest[,-(444:449)])
gbmPred2 <- predict(gbmFit2, dataTest[,-(444:449)])
svmPred <- predict(svmFitLin, dataTest[,-(444:449)])
svmPred2 <- predict(svmFitRBF, dataTest[,-(444:449)])
xgbPred <- predict(xgbFit, dataTest[,-(444:449)])
annPred <- predict(annFit, dataTest[,-(444:449)])


confusionMatrix(data = knnPred, reference = dataTest$Group)
confusionMatrix(data = ldaPred, reference = dataTest$Group)
confusionMatrix(data = plsPred, reference = dataTest$Group)
confusionMatrix(data = rfPred, reference = dataTest$Group)
confusionMatrix(data = gbmPred, reference = dataTest$Group)
confusionMatrix(data = gbmPred2, reference = dataTest$Group)
confusionMatrix(data = svmPred, reference = dataTest$Group)
confusionMatrix(data = svmPred2, reference = dataTest$Group)
confusionMatrix(data = xgbPred, reference = dataTest$Group)


# Stacking
# Stack the predictions from multiple models
stackPred <- data.frame(knnPred, ldaPred, plsPred,
                        gbmPred, rfPred, xgbPred,
                        svmPred, svmPred2, svmPred2,
                        class = dataTest$Group)
# Apply GBM to the stacked predictions
tic()
cl <- makeCluster(detectCores())
registerDoParallel(cl)
stackgbmFit <- caret::train(class ~ ., data = stackPred,
                            method = "gbm",
                            trControl = fitControl)
stopCluster(cl)
toc()
# Performance of the stacked model
stackgbmPred <- predict(stackgbmFit, stackPred[,-grep("class",colnames(stackPred))])
confusionMatrix(data = stackgbmPred, reference = stackPred[,grep("class",colnames(stackPred))])

# Resampling method - random for learning curve
ctrl1 <-trainControl(returnData = TRUE,
                     classProbs = TRUE,
                     summaryFunction = multiClassSummary)

# Resampling method - (group) replicate k fold
Repfolds <- groupKFold(data$Replicate, k = 3)
Randfolds <- groupKFold(data$Replicate, k = 3)
ctrl2 <- trainControl(index = Repfolds,
                      method = "cv",
                      returnData = TRUE,
                      classProbs = TRUE,
                      summaryFunction = multiClassSummary)


# Two resampling methods
######## 1 - Original sampling by group outcome - learning curve
knn_lc1 <- learning_curve_dat(dat = data[,-c(444,446:449)],
                              outcome = "Group",
```

```
                   # test_prob = 0.2,
                   verbose = TRUE,
                   method = "knn",
                   trControl = ctrl1)

# gbm
tic()
cl <- paralell::makeCluster(2, setup_strategy = "sequential")
registerDoParallel(cl)
gbm_lc1 <- learning_curve_dat(dat = data[,-c(444,446:449)],
                    outcome = "Group",
                    method = "gbm",
                    trControl = ctrl1,
                    verbose = FALSE)
stopCluster(cl)
toc()

# random forest
tic()
cl <- parallel::makeCluster(2, setup_strategy = "sequential")
registerDoParallel(cl)
rf_lc1 <- learning_curve_dat(dat = data[,-c(444,446:449)],
                   outcome = "Group",
                   method = "ranger",
                   trControl = ctrl1,
                   verbose = TRUE)
stopCluster(cl)
toc()

# SVM linear
tic()
cl <- parallel::makeCluster(4, setup_strategy = "sequential")
registerDoParallel(cl)
svmLin_lc1 <- learning_curve_dat(dat = data[,-c(444,446:449)],
                   outcome = "Group",
                   method = "svmLinear2",
                   trControl = ctrl1,
                   verbose = TRUE)
stopCluster(cl)
toc()


# SVM RBF
tic()
cl <- parallel::makeCluster(4, setup_strategy = "sequential")
registerDoParallel(cl)
svmRBF_lc1 <- learning_curve_dat(dat = data[,-c(444,446:449)],
                     outcome = "Group",
                     method = "svmRadialSigma")
stopCluster(cl)
toc()

# Plot
library(ggplot2)
ggplot(svmLin_lc1, aes(x = Training_Size, y = Accuracy)) +
  geom_smooth(method = loess, span = .8) +
  scale_y_continuous(breaks = seq(0.7,1,0.001))+
```

```
  scale_x_continuous(breaks = seq(800,9000,400))+
  theme_bw()


########## 2 - New resampling method by replicate
# kNN
tic()
cl <- parallel::makeCluster(4, setup_strategy = "sequential")
registerDoParallel(cl)
knn_rep <- train(Group ~ ., data = data[,-c(444,446:449)],
              method = "knn",
              trControl = ctrl3)
registerDoSEQ()
toc()

# GBM
tic()
cl <- parallel::makeCluster(4, setup_strategy = "sequential")
registerDoParallel(cl)
gbm_rep <- train(Group ~ ., data = data[,-c(444,446:449)],
              method = "gbm",
              trControl = ctrl2)
registerDoSEQ()
toc()

# Random Forest
tic()
cl <- parallel::makeCluster(4, setup_strategy = "sequential")
registerDoParallel(cl)
rf_rep <- train(Group ~ ., data = data[,-c(444,446:449)],
             method = "rf",
             trControl = ctrl2)
registerDoSEQ()
toc()

# SVM-linear
tic()
cl <- parallel::makeCluster(4, setup_strategy = "sequential")
registerDoParallel(cl)
svmLin_rep <- train(Group ~ ., data = data[,-c(444,446:449)],
             method = "svmLinear2",
             trControl = ctrl2)
registerDoSEQ()
toc()


# SVM-RBF
tic()
cl <- parallel::makeCluster(4, setup_strategy = "sequential")
registerDoParallel(cl)
svmRBF_rep <- train(Group ~ ., data = data[,-c(444,446:449)],
                method = "svmRadialSigma",
                trControl = ctrl2)
registerDoSEQ()
toc()

# Plot
```

```
trellis.par.set(caretTheme())
plot(svmRBF_rep)
svmLin_rep
```

SI References

(a) B. Brinkhof, et al., Improving characterisation of human Multipotent Stromal Cells cultured in 2D and 3D: Design and evaluation of primer sets for accurate gene expression normalisation. PLoS One 13, e0209772 (2018).

(b) H. Fukusumi, et al., Establishment of Human Neural Progenitor Cells from Human Induced Pluripotent Stem Cells with Diverse Tissue Origins. Stem Cells Int. 2016, 7235757 (2016).

(c) X. N. Wang, et al., Evaluation of the stability of reference genes in bone mesenchymal stem cells from patients with avascular necrosis of the femoral head. Genet. Mol. Res. 15, gmr7926 (2016).

(d) C. R. Muratore, P. Srikanth, D. G. Callahan, T. L. Young-Pearse, Comparison and optimization of hiPSC forebrain cortical differentiation protocols. PLoS One 9, e105807 (2014).

(e) E. W. Kuijk, et al., PTEN and TRP53 independently suppress Nanog expression in spermatogonial stem cells. Stem Cells Dev. 19, 979–988 (2010).

(f) A. Pouya, L. Satarian, S. Kiani, M. Javan, H. Baharvand, Human Induced Pluripotent Stem Cells Differentiation into Oligodendrocyte Progenitors and Transplantation in a Rat Model of Optic Chiasm Demyelination. PLoS One 6, e27925 (2011).

(g) S. Liedtke, J. Enczmann, S. Waclawczyk, P. Wernet, G. Kögler, Oct4 and its pseudogenes confuse stem cell research. Cell Stem Cell 1, 364–366 (2007).

(h) T. Palm, et al., Rapid and robust generation of long-term self-renewing human neural stem cells with the ability to generate mature astroglia. Scientific Reports 5, 16321 (2015).

(i) A. Ståhlberg, M. Bengtsson, M. Hemberg, H. Semb, Quantitative transcription factor analysis of undifferentiated single human embryonic stem cells. Clin. Chem. 55, 2162–2170 (2009).

(j) A. S. Haka, et al., Diagnosing breast cancer by using Raman spectroscopy. PNAS 102, 12371–12376 (2005).

(k) J. W. Chan, D. K. Lieu, T. Huser, R. A. Li, Label-Free Separation of Human Embryonic Stem Cells and Their Cardiac Derivatives Using Raman Spectroscopy. Anal. Chem. 81, 1324–1331 (2009).

(l) S. Rubina, M. Amita, D. Kedar K., R. Bharat, C. M. Krishna, Raman spectroscopic study on classification of cervical cell specimens. Vibrational Spectroscopy 68, 115–121 (2013).

(m) T. J. Harvey, et al., Classification of fixed urological cells using Raman tweezers. Journal of Biophotonics 2, 47–69 (2009).
